# Cellular uptake of modified mRNA occurs via caveolae-mediated endocytosis, yielding high protein expression in slow-dividing cells

**DOI:** 10.1016/j.omtn.2023.05.019

**Published:** 2023-05-20

**Authors:** Claudia Del Toro Runzer, Shivesh Anand, Carlos Mota, Lorenzo Moroni, Christian Plank, Martijn van Griensven, Elizabeth R. Balmayor

**Affiliations:** 1 Department of Cell Biology-Inspired Tissue Engineering, MERLN Institute for Technology-Inspired Regenerative Medicine, 6229 ER Maastricht, the Netherlands; 2 Department of Complex Tissue Regeneration, MERLN Institute for Technology-Inspired Regenerative Medicine, 6229 ER Maastricht, the Netherlands; 3 Ethris GmbH, 82152 Planegg, Germany; 4 Musculoskeletal Gene Therapy Laboratory, Rehabilitation Medicine Research Center, Mayo Clinic, Rochester, MN 55905, USA; 5 Experimental Orthopaedics and Trauma Surgery, Department of Orthopaedic, Trauma, and Reconstructive Surgery, RWTH Aachen University Hospital, 52074 Aachen, Germany

**Keywords:** MT: Delivery Strategies, gene therapy, plasmid DNA, chemically modified mRNA, lipid vectors, cellular uptake, endocytosis, transfection efficiency

## Abstract

Nucleic acids have clear clinical potential for gene therapy. Plasmid DNA (pDNA) was the first nucleic acid to be pursued as a therapeutic molecule. Recently, mRNA came into play as it offers improved safety and affordability. In this study, we investigated the uptake mechanisms and efficiencies of genetic material by cells. We focused on three main variables (1) the nucleic acid (pDNA, or chemically modified mRNA), (2) the delivery vector (Lipofectamine 3000 or 3DFect), and (3) human primary cells (mesenchymal stem cells, dermal fibroblasts, and osteoblasts). In addition, transfections were studied in a 3D environment using electrospun scaffolds. Cellular internalization and intracellular trafficking were assessed by using enhancers or inhibitors of endocytosis and endosomal escape. The polymeric vector TransIT-X2 was included for comparison purposes. While lipoplexes utilized several entry routes, uptake via caveolae served as the main route for gene delivery. pDNA yielded higher expression levels in fast-dividing fibroblasts, whereas, in slow-dividing osteoblasts, cmRNA was responsible for high protein production. In the case of mesenchymal stem cells, which presented an intermediate doubling time, the combination vector/nucleic acid seemed more relevant than the nucleic acid per se. In all cases, protein expression was higher when the cells were seeded on 3D scaffolds.

## Graphical abstract

**Figure:**
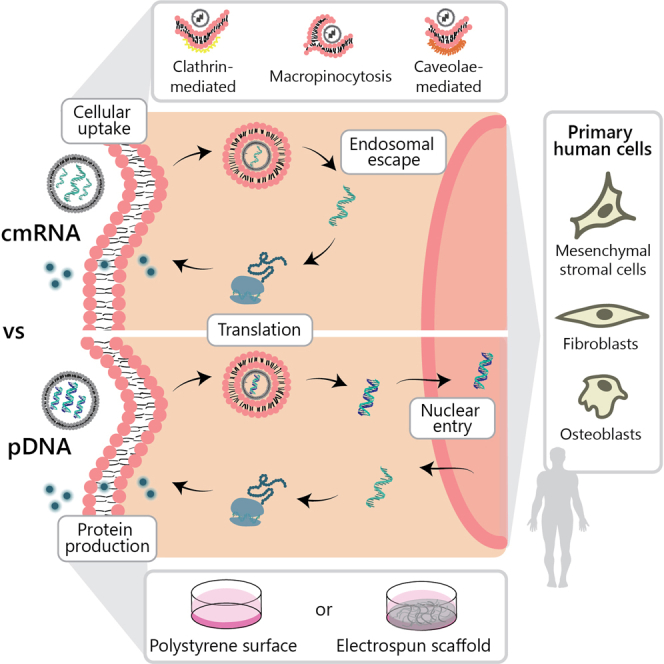

## Teaser

Balmayor and colleagues demonstrate that cellular uptake via caveolae-mediated endocytosis is the most productive route for delivery of mRNA and pDNA complexes. In slow-dividing cells, cmRNA yielded higher levels of expression compared with pDNA. This study provides insights into the choice of nucleic acid used in gene delivery for different cell types and culture scenarios.

## Introduction

Gene therapy is a method that uses nucleic acids to repair, replace, or regulate genes to prevent or treat pathological conditions.^1^ The delivery of nucleic acids presents several benefits over the direct administration of proteins. It allows the synthesis of one or more proteins with native conformation, true post-translational modifications, and has thus superior biological effect than recombinant proteins produced industrially.^2^^,^^3^ The industrial production of recombinant protein requires a large-scale cell line culture followed by extensive purification steps. The production procedures are different for each protein. This is not the case for nucleic acids, in which industrial production is simpler, straightforward, and highly reproducible. In addition, side effects associated with supraphysiological doses used in protein therapeutics are not expected with nucleic acid administration.

Delivery systems that transport genes of interest typically consist of two components. Those are nucleic acids with the genetic information encoding for the desired protein and a gene delivery material that will transport the nucleic acid across the cell membrane. Plasmid DNA (pDNA) was the first nucleic acid to be pursued as a therapeutic molecule and remains the most used for gene therapy. Later on, messenger RNA (mRNA) came into play as it offers several advantages. Unlike DNA, mRNA does not hold risks associated with genome integration.^4^^,^^5^ mRNA does not require transport across the nuclear membrane, it typically acts effectively upon release in the cytosol and is therefore functional in dividing and non-dividing cells. In addition, mRNA production is simpler and potentially more affordable than pDNA.^6^^,^^7^ Nonetheless, using mRNA also implies certain complications given its unstable nature and tendency to activate an innate immune reaction. Considerable efforts have been directed to tackle these issues by generating chemically modified mRNAs (cmRNAs), where structural elements are removed or replaced and modified nucleosides are used to circumvent the above-mentioned problems.^8^^,^^9^

To transport the therapeutic nucleic acid across the cell membrane a gene delivery material is often used. Lipids are one of the most commonly investigated non-viral vectors for gene delivery. They have been used in basic and biological research as well as preclinical and clinical investigations.^10^^,^^11^ Some of their advantages include their straightforward preparation, the possibility to tune several parameters to optimize transfection, and the lack of limitations regarding the size of their cargo.^12^

The therapeutic success of vectors and their cargo ultimately depends on the cellular uptake mechanism. It is generally accepted that both mRNA, as well as pDNA vectors, transfect cells via endosomal uptake.^13^^,^^14^ Endocytosis can be classified into two broad categories, phagocytosis, restricted to specialized cells, and pinocytosis, which occurs in all cell types.^15^ Pinocytosis can be further divided into macropinocytosis, clathrin-dependent endocytosis, and clathrin-independent endocytosis, among which, uptake from lipid rafts in caveolae is one of the most distinctive categories. Internalization of molecules via either endocytic method depends on multiple variables, including the size, charge, shape, surface chemistry, and presence of receptors, among others.^16^ Profiling cellular uptake is crucial for the efficient optimization of a gene delivery system. The cellular uptake mechanism will largely determine the intracellular processing of the internalized gene and its subsequent transfection efficiency. However, understanding the mechanistic insights of the endocytic pathway involved in the internalization of either pDNA or mRNA vectors also implies evaluating the vector properties and the cell type in question. These variables are all of crucial importance in the nucleic acid internalization outcome. Therefore, to study nucleic acid uptake mechanisms it is relevant to consider different types of gene carriers in an appropriate cell model.^17^

This paper seeks to address the differences in cellular uptake and transfection efficiencies between pDNA and mRNA complexes when transfecting primary cells of interest in the field of tissue engineering and regenerative medicine. Investigated cells included human mesenchymal stromal cells (hMSCs), human dermal fibroblasts (hDFs), and human osteoblasts (hOBs). Furthermore, we also aim to investigate transfection efficiencies of cells seeded on 2D monolayers and in a 3D microenvironment. These investigated conditions will lead to a better understanding of the potential uses of gene therapy for specific tissue engineering applications, emphasizing the importance of nucleic acid and carrier selection depending on the target cell and in a function-specific manner.

## Results

### Characterization of transfection complexes

**Figure 1:**
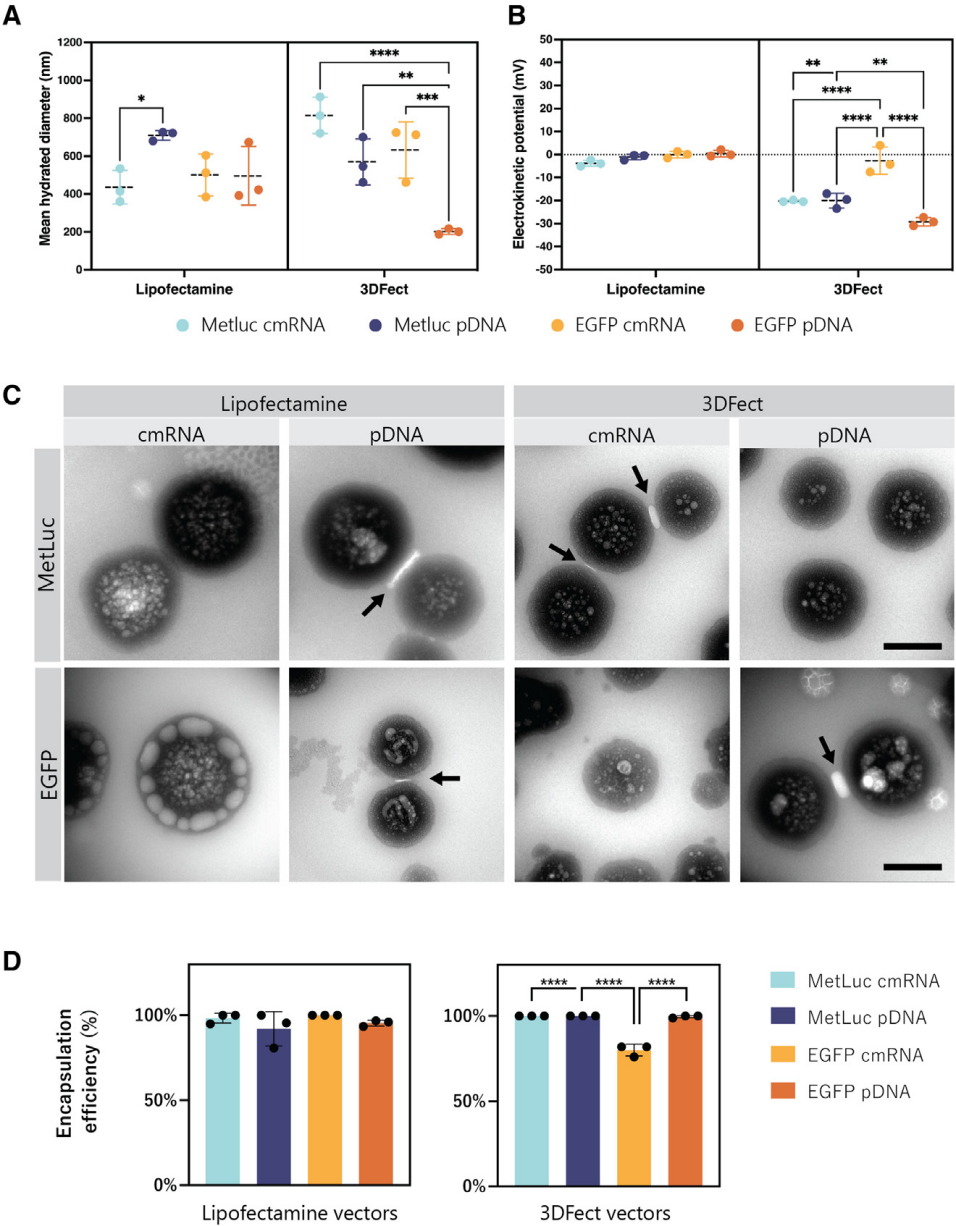
Characterization of cmRNA and pDNA lipid complexes (A) Size displayed as mean hydrated diameter and (B) electrokinetic potential of MetLuc and EGFP nucleic acid complexes. (C) Transmission electron microscopy photomicrographs show morphological features of the lipid complexes. Black arrows indicate bright, rod-like structures connecting the lipid complex particles. Scale bars, 250 nm. (D) Percentage of encapsulated MetLuc cmRNA, MetLuc pDNA, EGFP cmRNA, or EGFP pDNA using either Lipofectamine 3000 or 3DFect as lipid vector. Data are presented as mean ± SD (n = 3). Multiple comparisons were analyzed using two-way ANOVA with Sidak’s correction in (A and B), and one-way ANOVA followed by Tukey’s correction in (D). ∗p < 0.05, ∗∗p < 0.01, ∗∗∗p < 0.001, ∗∗∗∗p < 0.0001. MetLuc, Metridia luciferase; EGFP, enhanced green fluorescent protein.

Complexes formed with Lipofectamine 3000 showed a mean hydrated diameter in the range of 400–500 nm, except for Metridia luciferase (MetLuc) pDNA complexes, which featured average size of 709 ± 21 nm (Table S1). Interestingly, when compared with their cmRNA counterpart, MetLuc pDNA complexes were significantly larger in size (p = 0.0379) (Figure 1A). 3DFect complexes featured larger sizes that covered the range of 500–800 nm (Table S1). Surprisingly, 3DFect-EGFP (enhanced green fluorescent protein) pDNA complexes were the smallest of all complexes formulated in our study, featuring a diameter of 202 ± 12 nm. Interestingly, these complexes also showed the smallest polydispersity index (PdI), that is 0.21, indicative of an acceptable uniform sample with respect to the particle size (Table S1). The rest of the complexes studied here showed PdI values in the range of 0.29–0.38, indicating a mid-range polydispersity. Interestingly, complexes formed with the polymeric vector TransIT-X2 also showed some polydispersity, with PdI values of 0.4. TransIT-X2 complexes featured a mean hydrated diameter of 1,510 ± 47 nm for cmRNA and 1,760 ± 245 nm for pDNA (Table S1), making them the biggest complexes obtained in our study. Notably, Lipofectamine 3000 complexes featured an electrokinetic potential near 0 mV; that is, the complexes were neutrally or close to neutrally charged (Figure 1B). Conversely, the 3DFect complexes were characterized by a strong negative electrokinetic potential in the range of −20 to −30 mV. An exception was noted for the 3DFect-EGFP cmRNA complexes that featured an electrokinetic potential close to zero (−2.64 ± 4.8 mV, p < 0.0001) (Table S1; Figure 1B). Compellingly, polyplexes formed with TransIT-X2 showed a positive 16 ± 3.7 mV for cmRNA and 4.2 ± 0.1 mV for pDNA (Table S1). Worth mentioning is that complexation dramatically impacted the vectors' own charge; Lipofectamine 3000 featured an electrokinetic potential of −9.8 ± 3.4 mV that became zero while 3DFect’s 59.9 ± 3 mV converted to −18 ± 10 mV upon complexation. In the case of TransIT-X2 this change in charge was only noticed upon complexation with cmRNA. TransIT-X2 was characterized by an electrokinetic potential of 4.32 ± 0.83 mV that increased 4-fold in the cmRNA polyplexes.

Lipid complexes analyzed by transmission electron microscopy (TEM) revealed their morphological features (Figure 1C). During complexation, both lipids and nucleic acids underwent a complete topological transformation into compact, quasi-spherical particles. Both lipid vectors showed inner vesicles, suggesting an oligolamellar, multivesicular structure. Interestingly, in all the lipid complex formulations studied here, bright, rod-like structures were identified that connected the particles (black arrows in Figure 1C). Complexes formed by Lipofectamine 3000 and EGFP cmRNA showed distinguishable large vesicles in the outer layer, whereas smaller vesicles were found in the center. For the remaining complexes, medium size vesicles were found in the middle that seemed to decrease in size toward the periphery of the particles. The sizes of the complexes obtained by TEM was smaller than that obtained by DLS analysis. This is expected considering that hydrodynamic diameters are generally larger than the core nanoparticle sizes of a dried sample used for TEM. An ordered, multilamellar structure featuring periodic striations was only visible when ultrathin sections of the complexes were prepared (magenta arrows in Figure S1).

Both lipids, Lipofectamine 3000 and 3DFect were highly efficient in encapsulating cmRNA and pDNA. As depicted in Figure 1D, both nucleic acids were generally fully encapsulated. An exception was observed for 3DFect on the encapsulation of EGFP cmRNA. In this case, 80% encapsulation efficiency was obtained that was significantly lower when compared with all other 3DFect complexes (p < 0.0001). A non-significant reduction to 92% was observed for MetLuc pDNA vectors with Lipofectamine 3000 (p > 0.3).

### Endocytosis pathway determination

The inhibitors chlorpromazine, wortmannin, and genistein were selected to block the clathrin-mediated endocytosis, macropinocytosis, and caveolae-mediated endocytosis, respectively. Endocytosis inhibitors may feature cell-specific, concentration-dependent toxicity. To determine a concentration that results in low toxicity and favorable inhibition effect, the cytotoxicity of these inhibitors was assayed *in vitro* (Figure S2). Exposing hMSCs and hOBs to concentrations of chlorpromazine up to 28.1 μM (Figure S2A) showed metabolic activities comparable with the untreated group. Using higher concentrations resulted in a reduction on hMSCs’ metabolic activity by 14% (p = 0.0006), while hOBs increased their activity by 15% (p = 0.003). Notably, hDFs also showed increased metabolic activity upon contact with chlorpromazine. It remained higher than the metabolic activity of control cells for the entire range of chlorpromazine concentrations tested (p < 0.05). Metabolic activities of the cells in contact with wortmannin (Figure S2B) remained unaltered for concentrations up to 0.05 μM. At higher concentrations, hMSCs and hDFs reduced their metabolic activity to 83% (p < 0.0001) and 90% (p > 0.05), respectively. Treatment with genistein (Figure S2C) resulted in a 40% reduction of metabolic activity of the hDFs starting from the lowest concentration evaluated (i.e., 100 μM, p < 0.0001). Conversely, hOBs and hMSCs seemed to be unaffected by the treatment with this inhibitor at concentrations in the range 100–350 μM (p > 0.23). On the basis of these data, concentrations of 28.1 μM chlorpromazine, 0.05 μM wortmannin, and 200 μM genistein were selected to perform the inhibition experiments.

**Figure 2:**
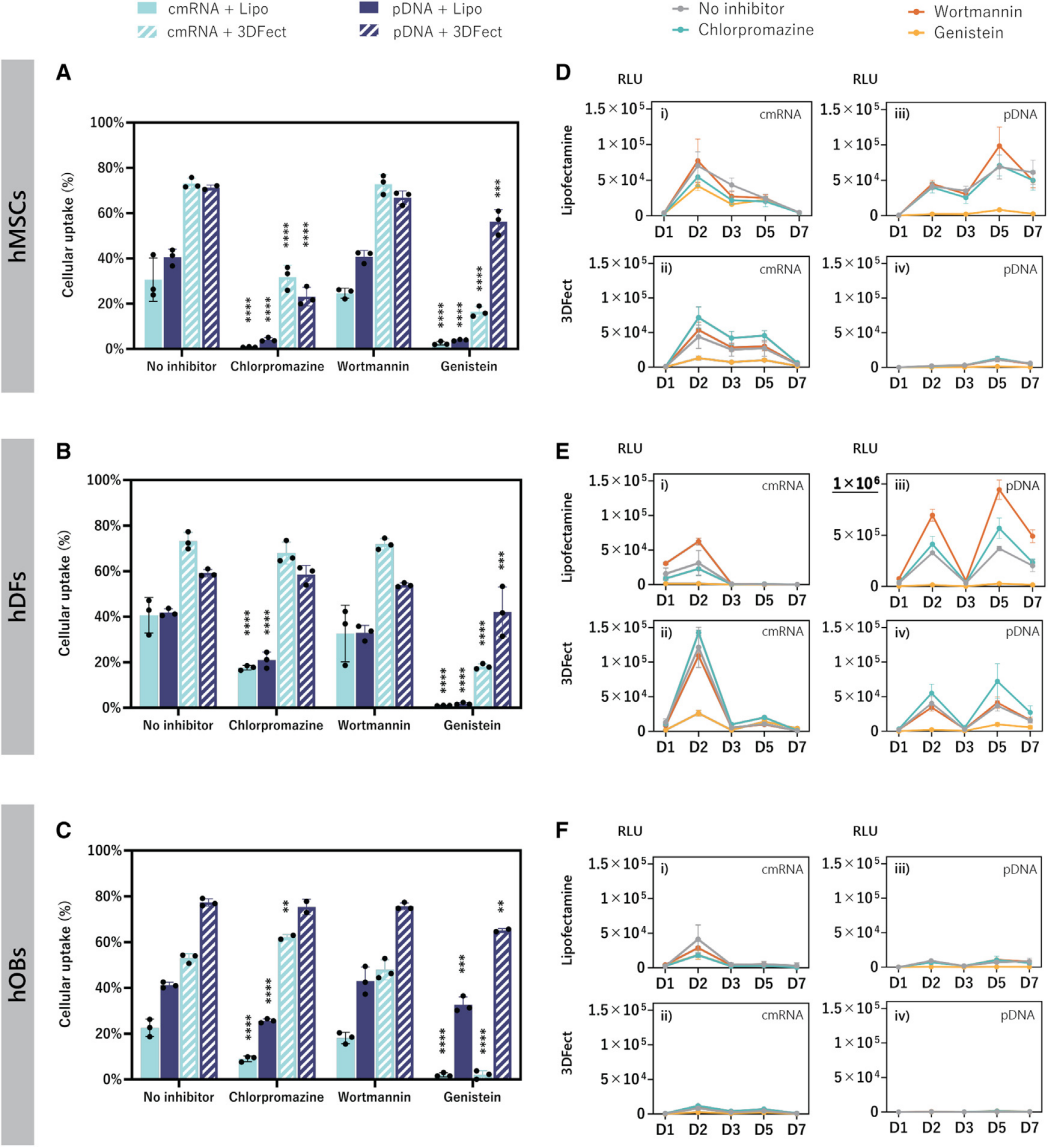
Effect of endocytosis inhibitors on cellular uptake of MetLuc cmRNA and MetLuc pDNA lipoplexes Percentages of intracellular uptake of MFP488-labeled MetLuc complexes before and after inhibition using chlorpromazine, wortmannin, or genistein, as determined by flow cytometry for (A) hMSCs, (B) hDFs, and (C) hOBs. Transfection efficiencies were determined by luminescent measurements after transfection in the presence of the inhibitors of (D) hMSCs, (E) hDFs, and (F) hOBs, using complexes of (i) MetLuc cmRNA with Lipo, (ii) MetLuc cmRNA with 3DFect, (iii) MetLuc pDNA with Lipo, and (iv) MetLuc pDNA with 3DFect. Data are presented as mean ± SD (n ≥ 3). Multiple comparisons were analyzed using two-way ANOVA with Dunnett’s correction. ∗∗p < 0.01, ∗∗∗p < 0.001, ∗∗∗∗p < 0.0001. MetLuc, Metridia luciferase; hMSCs, human mesenchymal stromal cells; hDFs, human dermal fibroblasts; hOBs, human osteoblasts; Lipo, Lipofectamine 3000; RLU, relative light units. Of note, the y axis in (E) (iii) differs from the other graphical representations by going up to 1 × 10^6^ RLU.

Percentage of cellular uptake, before and after endocytosis inhibition, was determined by flow cytometry. Without an inhibitory effect, cells transfected with 3DFect complexes yielded significantly higher uptake (72.4% for hMSCs, 66.2% for hDFs, and 65.1% hOBs) than the cells treated with Lipofectamine 3000 complexes (35.6% for hMSCs, 41.2% for hDFs, and 31.8% hOBs) (Figures 2A–2C) (p ≤ 0.0008 for all comparisons). The superiority of 3DFect over Lipofectamine 3000 regarding nucleic acid internalization was independent of the cell type and the nucleic acid used.

In hMSCs (Figure 2A), blockage of clathrin-dependent endocytosis using chlorpromazine strongly interfered with cellular uptake of all the complexes (reduction of 33% for Lipofectamine 3000 and of 45% for 3DFect, p < 0.0001), whereas in hDFs and hOBs (Figures 2B and 2C) cellular uptake was reduced significantly only for Lipofectamine 3000 complexes to 22% and 14%, respectively (p < 0.0001). Curiously, the hOBs uptake of 3DFect complexes formed with cmRNA slightly increased in 3% upon chlorpromazine treatment (Figure 2C). Regardless of the cell type and nucleic acid used, wortmannin treatment for disruption of macropinocytosis did not show a noteworthy effect on cellular uptake of the lipid complexes. Conversely, blockage of caveolae-mediated endocytosis with genistein in hMSCs and hDFs significantly reduced cellular uptake of Lipofectamine 3000 complexes to near zero values (p < 0.0001) (Figures 2A and 2B). This effect was independent of the type of nucleic acid used. Internalization of 3DFect complexes was also impaired by genistein, although, in this case, a noticeable difference was observed between pDNA and cmRNA complexes. Cellular uptake of 3DFect cmRNA decreased to 55% in hMSCs and hDFs after treatment with genistein (p < 0.0001). The reduction of 3DFect pDNA cellular uptake by hMSCs was of 17% (p < 0.001). Remarkably, in hOBs, genistein showed stronger inhibitory effect for lipid complexes containing cmRNA (uptake reduced to 2%) than pDNA (uptake reduced to 30%), regardless of the type of lipid used (Figure 2C) (p < 0.0001).

MetLuc protein expression upon cellular uptake inhibition was assessed to better understand the effect of impaired nucleic acid internalization on protein production. Results in Figures 2D–2F confirmed that genistein led to an effective suppression of protein expression that was visible in all three cell types and for all complexes used. Conversely and in disagreement with previous cellular internalization data, treatments with chlorpromazine (Figures 2Dii and 2Eii, iii, iv) and wortmannin (Figures 2Di, ii, iii and 2Ei, iii) did not lead to any reduction, but rather to an increase on protein production. An exception was observed for Lipofectamine 3000 cmRNA complexes. In this case, inhibition of cellular internalization with chlorpromazine negatively impacted MetLuc expression (Figures 2D–2Fi).

### Cellular internalization of complexes

**Figure 3:**
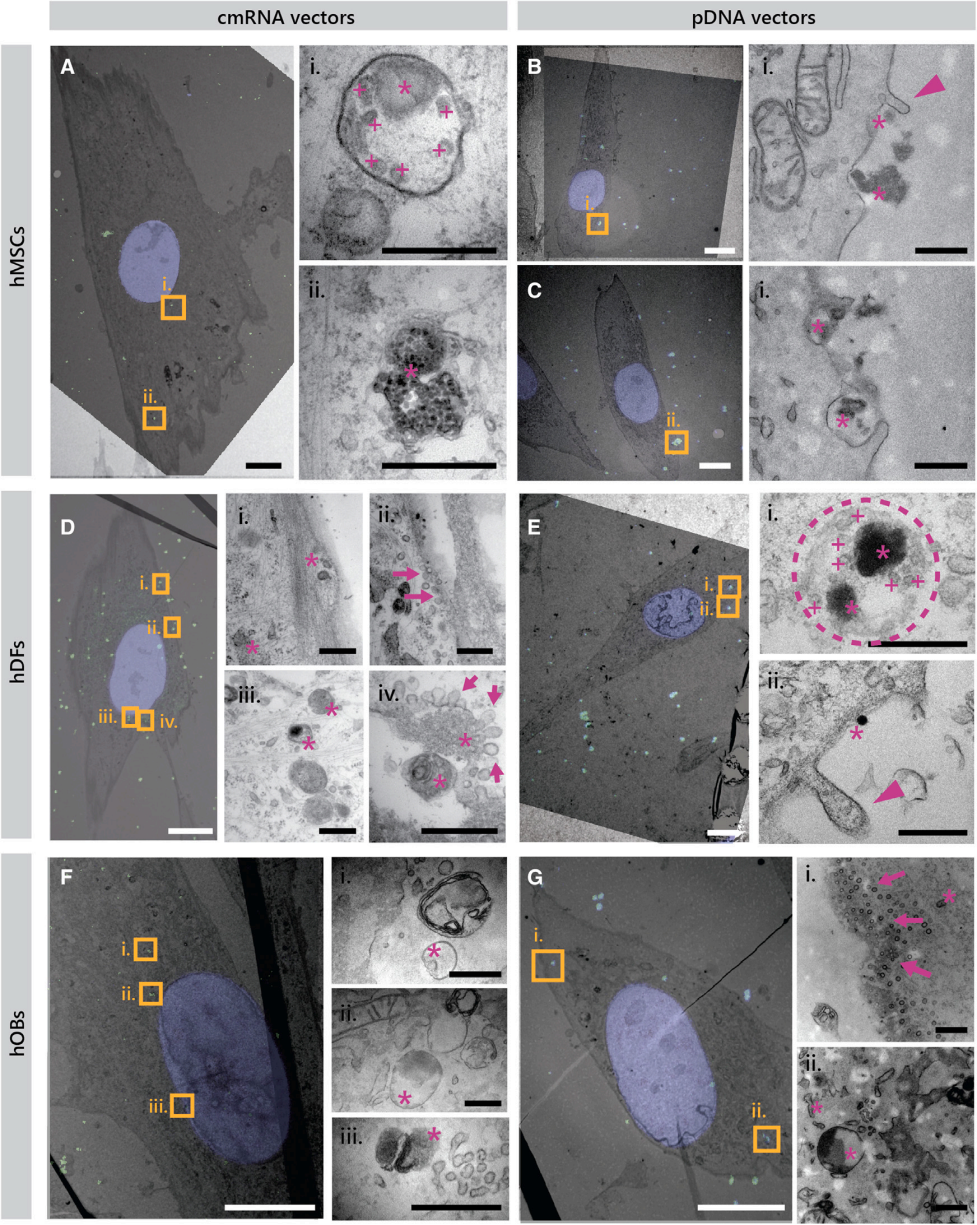
Correlative light electron microscopy of cells transfected with MFP-488-labeled MetLuc cmRNA or MFP-488-labeled MetLuc pDNA using Lipofectamine 3000 vectors Photomicrograph in (A–C) illustrate hMSCs, (D and E) hDFs, and (F and G) hOBs. Nuclear staining with Hoechst is shown in blue and MFP-488-labeled lipoplexes are visible in green. Images in (i), (ii), (iii), and (iv) resulted from ultrathin sections and represent high-magnified regions of interest framed in yellow in the correlated image. Magenta asterisks depict the specific localization of the fluorescent complexes. Magenta arrows point toward small flask-shaped invaginations of the plasma membrane, characteristic of caveolae. Magenta triangles point toward extensions of plasma membrane ruffles typically present during macropinocytosis. A dotted circle in (E) (i) surrounds a late endosome loaded with lipid complexes. Magenta plus symbols indicates the intraluminal vesicles inside the endosome. Scale bars, 10 μm (A–G) and 500 nm (i, ii, iii, and iv) (images of the magnified areas). MetLuc, Metridia luciferase; hMSCs, human mesenchymal stromal cells; hDFs, human dermal fibroblasts; hOBs, human osteoblasts.

**Figure 4:**
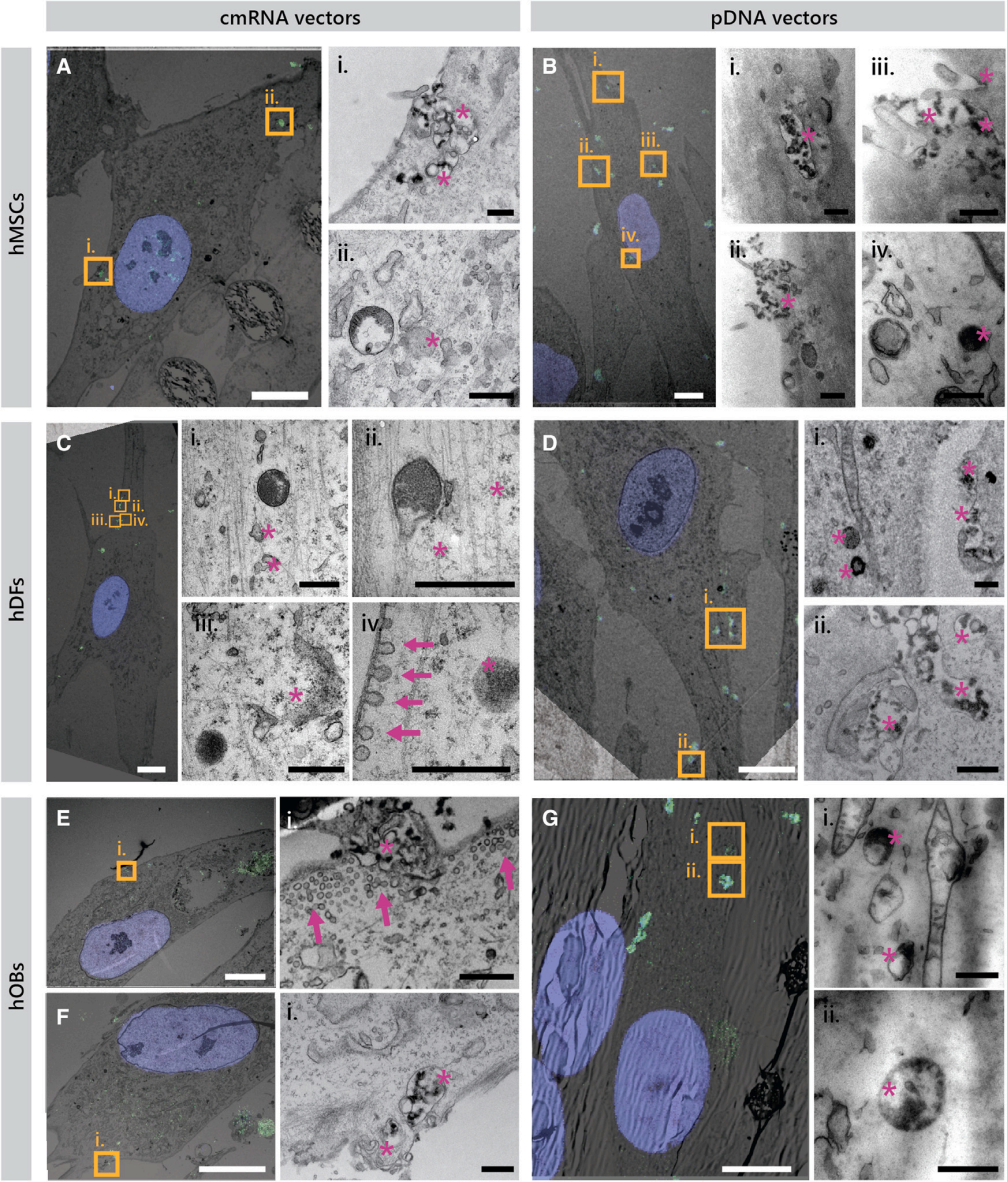
Correlative light electron microscopy of cells transfected with MFP-488-labeled MetLuc cmRNA or MFP-488-labeled MetLuc pDNA by means of 3DFect vectors Photomicrograph in (A and B) illustrate hMSCs, (C and D) hDFs, and (E–G) hOBs. Nuclear staining with Hoechst is shown in blue and MFP-488-labeled lipoplexes are visible in green. Images in (i), (ii), (iii), and (iv) resulted from ultrathin sections and represent high-magnified regions of interest framed in yellow in the correlated image. Magenta asterisks depict the specific localization of the internalized fluorescent complexes. Magenta arrows point toward small flask-shaped invaginations of the plasma membrane, characteristic of caveolae. Scale bars, 10 μm (A–G) and 500 nm (i, ii, iii, and iv) (images of the magnified areas). MetLuc, Metridia luciferase; hMSCs, human mesenchymal stromal cells; hDFs, human dermal fibroblasts; hOBs, human osteoblasts. A corresponding video is available as Video S1 showing the trafficking of mRNA complexes to lysosomal compartments in human osteoblasts.

Fluorescent images were overlapped with transmission electron photomicrographs of the exact same cell to visualize the cellular uptake of the lipid complexes by the different cell types studied here (Figures 3A–3G for Lipofectamine 3000 and Figures 4A–4G for 3DFect). This technique allows the identification of fluorescently labeled nucleic acid-complexes at the ultrastructural level within different cellular compartments. The selection of the analyzed regions of interest (ROIs) from a general image has been illustrated in Figures S3–S5 for hMSCs, hDFs, and hOBs respectively.

At 1 h after transfection, several lipid complexes have entered the cells as they were observed located in the cytosol (e.g., Figures 3Diii, 3Fiii, and 4Gii), while some others appeared to be initiating the process of internalization as they are located in the proximity of the cell membrane (e.g., Figures 3Bi, 3Dii, and 4Fi). Aggregated complexes are also distinguishable that seem to be internalized at once forming cell membrane invaginations (Figures 3Aii, 3Bi, 4Ai, 4Bi–iii, and 4Dii). These complexes were mainly localized within cell membrane-bound structures and as endocytic vesicles. Some of the vectors bound to the cell membrane were associated with flask-shaped invaginations that resembled caveolae, typically located in lipid rafts (Figures 3Dii, iv, 3Gi, 4Cii, and 4Ei). In some cases, individual caveosomes might be too small to internalize some of the lipid complexes. This could lead to the fusion of several caveolae to successfully internalize lipid complexes. Such caveosomes can be observed in Figure S6, where they appear as rosette-shaped structures (magenta arrows). Complexes within invaginations and endosomes (magenta asterisks in Figures 3Ci, and 3Ai, respectively) were identified, which may indicate the presence of clathrin receptors. In the latter, as in Figures 3Ei, more than six intraluminal vesicles depicted with magenta plus symbols, can be further visualized. This indicates the maturation of these bodies into late endosomes. In addition, complexes were also present in areas of the cell membrane that lacked invaginations or lipid rafts. Instead, these areas presented cellular protrusions that resemble macropinosomes (magenta triangles in Figures 3Bi, and 3Eii). Specifically in hOBs, “giant endosomes” were observed (Figures S7A and S7B) exceeding several micrometers in diameter with heterogeneous content that included fluorescently labeled lipid complexes. This might imply that osteoblasts are also internalizing the lipid complexes through phagocytosis. Furthermore, large, dense structures resembling lysosomes were also observed in hOBs (Figure S7C and S7D).

Some of the internalized nucleic acids were found free in the cell cytoplasm and next to degraded vectors or endosomes with dissolving membranes (Figures 3Fi, ii, 4Aii, and 4Ci–iii). Notably, no pDNA complex was found located in the nucleus at this time of observation. In hMSCs, however, pDNA complexes were found located in the vicinity of the cell nuclei (Figures 4Biv). A common feature observed in all cell types investigated was mitochondria located in close proximity to the internalized endosomes containing complexes (e.g., Figure 4Di, 4Gi, S3Biii, S4Bi, and S4Di).

### Endosomal and lysosomal trafficking of internalized complexes

To further investigate intracellular trafficking of the complexes after internalization, cells were treated with drugs that either enhanced or inhibited endosome release, i.e., chloroquine or bafilomycin, respectively (Figure S8). Chloroquine is a lysosomotropic agent that promotes rupture of endosomes. Interestingly, at day 1 post-transfection, pre-treatment with chloroquine increased MetLuc activity in most conditions. This increment was significant in hDFs and hOBs upon transfection with MetLuc cmRNA and Lipofectamine 3000 (Figures S8B and S8C, p < 0.0001). Unexpectedly, the same enhancement of transfection efficiency was not noted for hDFs transfected with pDNA-Lipofectamine 3000. In this case, pre-treatment with endosomal escape enhancer chloroquine negatively impacted the transfection efficiency (pDNA graph in Figure S8B). Of note, the effect of the endosomal escape enhancer drug was observed only at day 1. Later, from day 2 to 7, the effect of the drug was weakened and a higher MetLuc activity was observed in the groups with no drug pre-treatment. Conversely, pre-treatment with endosomal release inhibitor bafilomycin irreversibly and significantly reduced MetLuc activity under all conditions (Figure S8). The drastic decrease in protein production by all the cell types, regardless of the nucleic acid or vector used, indicated the effectivity of bafilomycin in inhibiting endosomal release. Notably, the short, initial endosomal escape inhibition resulted in a prolonged (over 7 days) reduction of protein production.

LysoTracker Deep Red labeling of the acidic lysosomes showed a punctate staining pattern in all three cell types transfected in the absence of endosomal enhancer or inhibitor drug or upon treatment with chloroquine (Figures S9 and S10). At 3 h post-transfection, colocalization of MFP-488 fluorescently labeled complexes with stained lysosomes was possible using high-resolution confocal microscopy (no drug, Figure S9Ai–vi and Figure S10Ai–v). Distinct colocalized complexes appear in yellow from the overlap of the red lysosomes and green complexes in the zoomed-in images (Figures S9Bi–vi and S10Bi–v). While several complexes overlapped with lysosomes when no enhancer or inhibitor drug was used, with chloroquine treatment it was only possible to colocalize one labeled Lipofectamine 3000 complex with lysosomes in hOBs (Figures S9A and S9Bvii). Notoriously, pre-treatment with bafilomycin suppressed staining of lysosomes given that this dye accumulates in acidic compartments while the drug largely de-acidifies lysosomes. Moreover, a consistent reduction on the number of Lipofectamine 3000 complexes (Figure S9) was observed when compared with the TransIT-X2 complexes (Figure S10) in the bafilomycin-treated groups. A distinctive number of complexes accumulating in lysosomes can be appreciate in the time-lapse video of hOBs transfected with 3DFect (Video S1).

Video S1. Trafficking of complexes to lysosomal compartments Two-hour time-lapse video of internalized of 3DFect complexes colocalized with lysosomes in human osteoblasts. LysoTracker Deep Red-stained acidic compartments in red, nuclear staining with Hoechst is shown in blue, and MFP-488-labeled MetLuc complexes in green

### ES scaffold characterization

**Figure 5:**
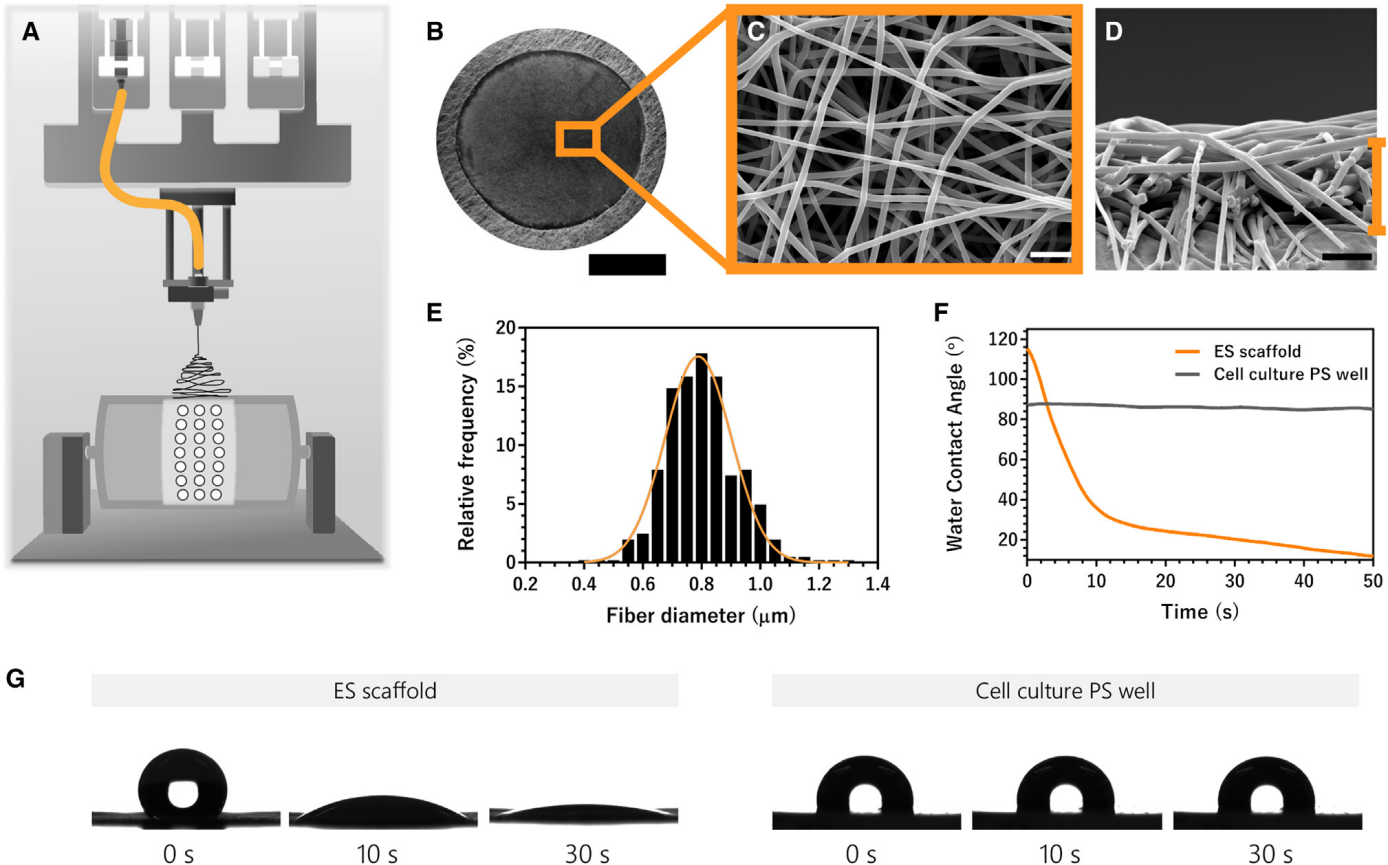
3D, ES scaffold fabrication and characterization (A) Schematic of the electrospinning setup. (B) Stereomicroscope image of the complete ES scaffold designed to fit in 24-well plates. Scanning electron microscopy micrograph highlighting (C) the electrospun nanofibers with an average diameter of 0.792 ± 0.138 μm and (D) the cross-section that indicates a scaffold thickness of 5 μm. (E) Relative frequency distribution analysis of the corresponding fiber diameters, demonstrating a balanced Gaussian curve (orange). (F) Water contact angle measurements over an extended period of time comparing the ES scaffold with the standard culture plate. (G) Pictures of water droplets over time on ES scaffold and cell culture polystyrene wells. Scale bars, 5 mm (B) and 5 μm (C and D). ES, electrospun; PS, polystyrene; s, second.

Scanning electron microscopy (SEM) micrographs of the fabricated ES scaffolds (Figures 5A and 5B) revealed an average fiber diameter of 0.792 ± 0.138 μm (Figure 5C) along with a cross-sectional thickness of 5 μm (Figure 5D). Moreover, the frequency histogram of the obtained diameters demonstrated a homogeneous distribution of the collected fibers within each scaffold (Figure 5E). To evaluate the hydrophilicity of the ES scaffolds, the water contact angle (WCA) profile was determined over a period of 50 s. The ES scaffolds started with a WCA of 117°; however, their porous morphology allowed the angle to drop to 35° within 10 s (Figure 5F). In contrast, the polystyrene well plate (used as a surface for the 2D cell monolayer generation) showed a consistent WCA of 86.5° throughout 50 s, thereby validating the superior hydrophilic behavior of the ES scaffolds with respect to the polystyrene surface of the cell culture well plate (Figure 5G).

### Transfection efficiency of cmRNA and pDNA in 2D and 3D cultures

**Figure 6:**
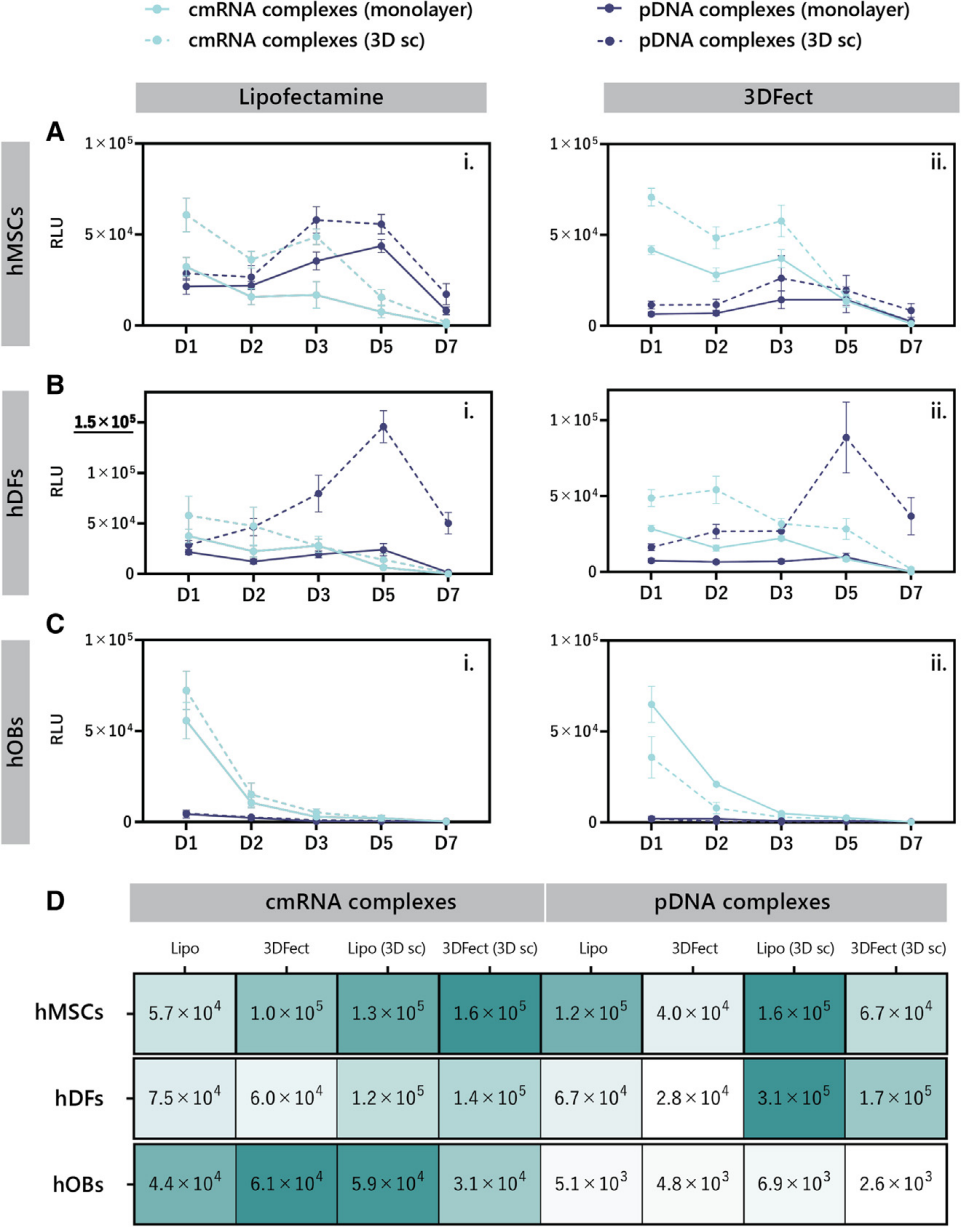
MetLuc expression over time for cells seeded on standard cell culture wells or 3D, ES scaffolds Transfection efficiency is indicated as RLU over a time course of 7 days in (A) hMSCs, (B) hDFs, and (C) hOBs. Cells were transfected with either cmRNA (light blue lines) or pDNA (dark blue lines) both encoding for MetLuc. Continuous lines indicate cells cultured on standard cell culture wells and dotted lines indicate cells cultured on 3D, ES scaffolds. Lipofectamine 3000 results are depicted in (i) graphs, while 3DFect results are in (ii) graphs. Data are presented as mean ± SD (n = 5). Multiple comparisons were analyzed using two-way ANOVA with Sidak’s correction. Of note, the y axis in (B) (i) differs from the other graphical representations by going up to 1.5 × 10^5^ RLU. (D) Heatmap analysis of the AUC values of the graphs presented in (A–C) calculated by integrating the data between zero and day 7 post-transfection. ES, electrospun; RLU, relative light units; hMSCs, human mesenchymal stromal cells; hDFs, human dermal fibroblasts; hOBs, human osteoblasts; MetLuc, Metridia luciferase; AUC, the area under the curve; Lipo, Lipofectamine 3000.

Remarkable differences on protein expression were observed between cell types, and 2D or 3D seeding environment. Early MetLuc expression, that is at day 1 post-transfection, was appreciated in hMSCs transfected with cmRNA complexes. Unexpectedly, a decrement on MetLuc expression was observed at day 2 to then increase again at day 3 post-transfection (Figure 6A). Complexes containing pDNA showed later onsets of expression, with peaks between days 3 and 5 post-transfection. Comparing the 2D with the 3D environment, a higher MetLuc expression was obtained when hMSCs were seeded on the 3D, ES scaffolds (dotted lines in Figures 6Ai, ii), which was independent of the type of nucleic acid used. Analysis of the area under the curve (AUC) of the MetLuc expression graphs (Figure 6D) indicated a maximal protein expression when hMSCs were transfected on 3D, ES scaffolds loaded with either 3DFect cmRNA or Lipofectamine 3000 pDNA complexes. Interestingly, complexes of 3DFect with pDNA seemed to be inefficient in transfecting hMSCs monolayers.

Expression kinetics of MetLuc in hDFs (Figure 6B) revealed a prominent difference between nucleic acids and lipids. Higher protein expression was generally observed for pDNA transfections performed on 3D scaffolds. A peak of expression was detected at day 5 post-transfection. cmRNA transfections performed on the 3D scaffolds showed maximum protein expression at early time of observation with a subsequent decay from day 3 on. Both Lipofectamine 3000 and 3DFect complexes resulted in low MetLuc expression, below 5 × 10^4^ RLU (relative light units) when transfected into hDFs monolayers (Figure 6B). Overall, an enhancement of the transfection efficiency of both lipids was observed when hDFs were transfected in a 3D environment (Figures 6B and 6D).

Remarkably, transfection of hOBs was only successful via cmRNA nucleic acid complexes (Figure 6C). From day 2 post-transfection onward, levels of protein expression showed no dependence on the lipid vector used (p ≥ 0.9 for monolayers and p > 0.3 for 3D scaffolds). Opposite to hDFs, pDNA complexes showed a very poor expression when transfected into hOBs, with a 10-fold reduction of the AUC values (Figure 6D). Surprisingly, cmRNA transfection of hOBs seeded on the 3D scaffolds failed to show the marked improvement on protein expression previously concluded for hMSCs (Figure 6Ci).

These results were correlated with cellular growth parameters for each cell type (Table S2). Cells with the shortest doubling time (i.e., hDFs with 1.11 days, fast multiplying cells) seemed to be easier to transfect using pDNA lipid complexes, whereas transfection of cells with the longer doubling time (i.e., hOBs with 9.63 days, slow multiplying cells) only seemed to be effectively transfected by cmRNA lipid complexes. hMSCs, which have a doubling time of 2.05 days, seem to be equally easy to transfect by either nucleic acid.

### Cytotoxicity of lipids, nucleic acids, and transfection complexes

3DFect showed to be generally less toxic than Lipofectamine 3000 for hMSCs and hOBs. Interestingly, hOBs appeared to be more susceptible to Lipofectamine 3000 than hMSCs. Lipofectamine 3000 toxicity for hMSCs was significant only at day 1 post-treatment (p = 0.0086, Figure S11Ai), while the toxicity of this lipid for hOBs was constant over the entire time frame studied (p = 0.0032 for day 1, p = 0.01 for day 3, and p = 0.0337 for day 7 post-treatment, Figure S11Ci). Conversely, hMSCs showed to be more susceptible to the action of naked nucleic acids (Figure S11Aii). Naked pDNA was significantly more toxic than naked cmRNA for up to 7 days of treatment (p = 0.04 for day 1, p = 0.0026 for day 3, and p = 0.0184 for day 7 post-treatment, Figure S11Aii). Transfection of all three cell types showed to be generally less toxic with cmRNA when compared with pDNA lipid complexes (Figures S11A, S11B, and S11Ciii, iv). Transfection of hMSCs with pDNA complexes generated a cytotoxic effect indicated by the gradual reduction of the cellular metabolic activity (p = 0.0012 for day 1, p = 0.01 for day 3, and 0.0012 for day 7 post-transfection, Figure S11Aiii). This was independent of the lipid vector used. In fact, similar toxicity pattern was observed for both, 3DFect pDNA and Lipofectamine 3000 pDNA complexes (p < 0.0001 for day 1, p = 0.0006 for day 3, and 0.0001 for day 7 post-transfection, Figure S11Aiv). Although pDNA lipid complexes were also more toxic than the cmRNA ones, this effect was less pronounced for hDFs when compared with hMSCs, and only significant from day 3 post-transfection onward (p > 0.05 for day 1, and p ≤ 0.003 for days 3 and 7 post-transfection, Figure S11Biii, iv). When analyzing hOBs, significant toxicity of pDNA lipid complexes was concluded for both lipid vectors at day 7 post-transfection (p = 0.0264 for Lipofectamine 3000 and p = 0045 for 3DFect, Figure S11Ciii, iv). Noteworthy, 3DFect cmRNA lipid complexes showed to be highly biocompatible up to 7 days post-transfection of hOBs (Figure S11Civ).

Performing transfections in 3D scaffolds further improved the biocompatibility of cmRNA lipid complexes for hMSCs (Lipofectamine 3000, p = 0.0088 at day 1; and 3DFect, p = 0.0458 at day 1 and p = 0.024 at day 3 post-transfection, Figure S11Aiii, iv). Interestingly, the same was not observed for pDNA transfections of hMSCs (p > 0.05, Figure S11Aiii, iv). Similarly, no improvement in the biocompatibility of cmRNA and pDNA lipid complexes was observed in hDFs (Figure S11Biii, iv) or hOBs (Figure S11Ciii, iv) upon transfections in 3D scaffolds (p > 0.05).

### EGFP transfection

**Figure 7:**
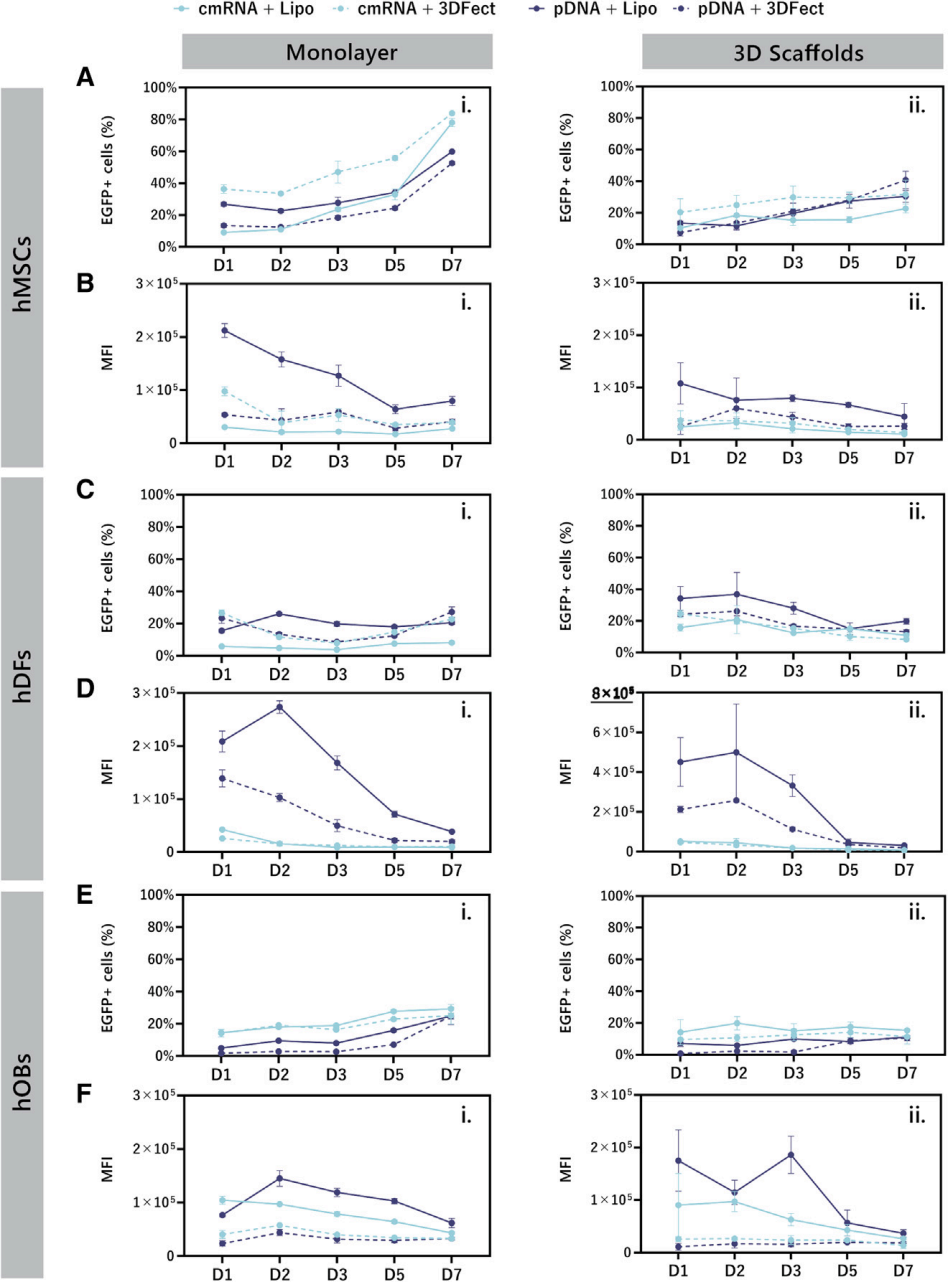
Enhanced green fluorescent protein expression over time for cells seeded on standard cell culture wells or 3D, ES scaffolds as determined by flow cytometry Percentage of EGFP-positive (A) hMSCs, (C) hDFs, and (E) hOBs plotted as normalized values to a total cell number. Mean fluorescence intensity of EGFP positive events for transfected (B) hMSCs, (D) hDFs, and (F) hOBs. Cells were transfected on (i) monolayers or (ii) 3D, ES scaffolds. Cells were transfected with either cmRNA (light blue lines) or pDNA (dark blue lines) both encoding for EGFP. Continuous lines and dotted lines indicate complexes were made with Lipofectamine 3000 or 3DFect, respectively. Data are presented as mean ± SD (n = 3). Multiple comparisons were analyzed using two-way ANOVA with Tukey’s correction. Of note, the y axis in (D) (ii) differs from the other graphical representations by going up to 8 × 10^5^ MFI. ES, electrospun; EGFP, enhanced green fluorescent protein; hMSCs, human mesenchymal stromal cells; hDFs, human dermal fibroblasts; hOBs, human osteoblasts; MFI, mean fluorescence intensity; Lipo, Lipofectamine7 3000.

**Figure 8:**
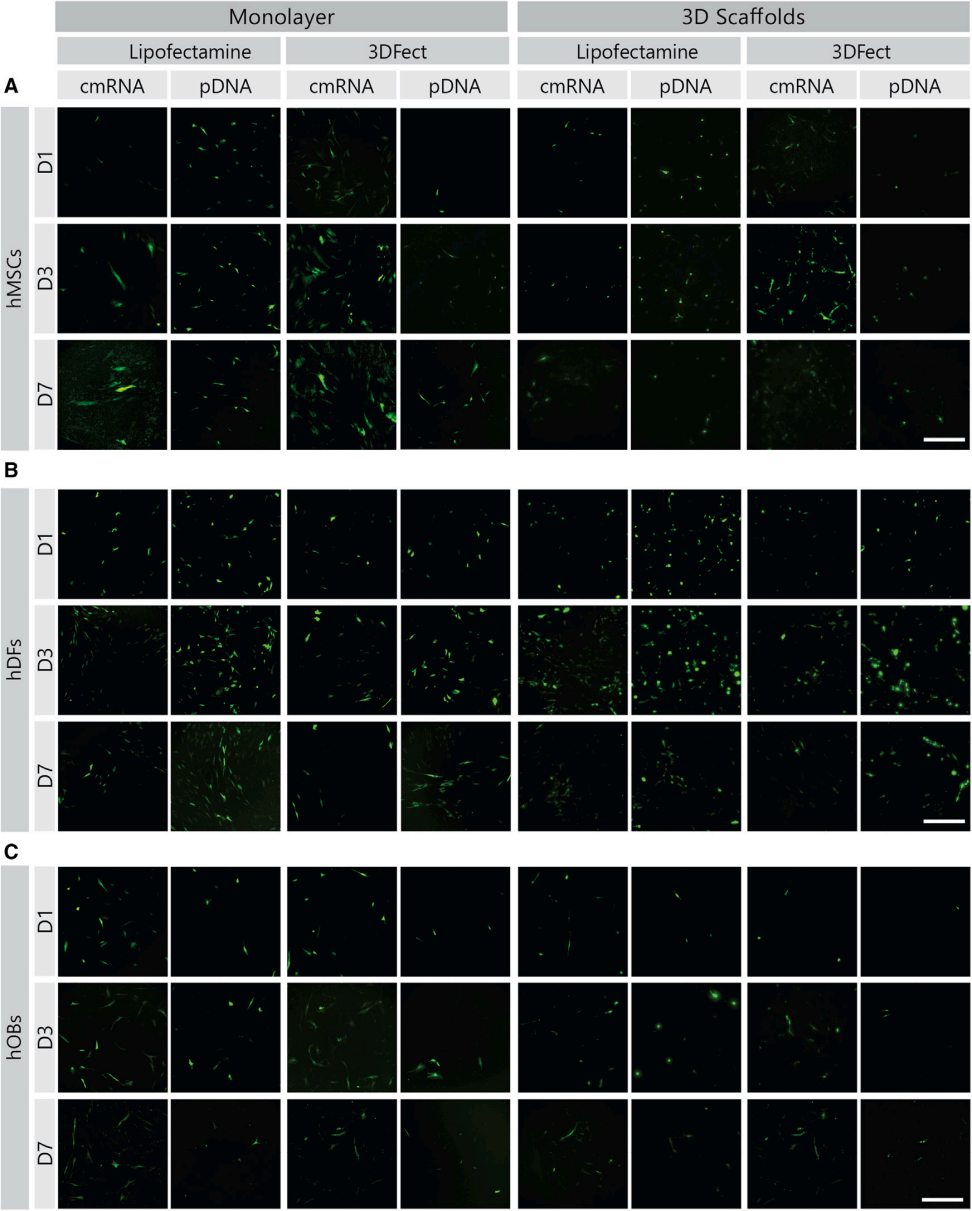
Enhanced green fluorescent protein expression over time for cells seeded on standard cell culture wells or 3D, ES scaffolds as imaged by fluorescence microscopy Representative images of the cells were taken on days 1, 3, and 7 after transfection with either cmRNA or pDNA encoding for EGFP using Lipofectamine 3000 or 3DFect as delivery vectors. The left section of the panel corresponds to cells transfected in monolayers that were formed on standard cell culture plates. The right section of the panel corresponds to cells transfected on 3D, ES scaffolds. Images correspond to (A) hMSCs, (B) hDFs, and (C) hOBs. Scale bars, 500 μm. EGFP, enhanced green fluorescent protein; ES, electrospun; hMSCs, human mesenchymal stromal cells; hDFs, human dermal fibroblasts; hOBs, human osteoblasts; D, day.

The percentage of EGFP-expressing cells and the relative amount of transgene per cell, expressed as mean fluorescence intensity (MFI) of EGFP-positive cells is shown in Figure 7. In addition, fluorescent microscopy pictures were taken to complement the quantitative data and are depicted in Figure 8. 3DFect appeared to be favorable for cmRNA transfections of hMSCs while Lipofectamine 3000 gave better results with pDNA (Figures 7Ai, ii). This was particularly noticeable for transfections of cell monolayers. Transfections in a 3D environment yielded a maximum of 40.8% ± 5.5% EGFP-positive cells in contrast to 83.4% ± 0.3% EGFP-positive cells obtained in monolayers. Remarkably, no peak with subsequent decay was observed during hMSC transfection. The percentage of positive cells steadily increased up to day 7 post-transfection of cell monolayers. Conversely, no clear increase of EGFP-positive cells could be concluded for hMSC transfections on 3D scaffolds. As for hMSCs, transfection of hDFs monolayers with cmRNA was more efficient by means of 3DFect (Figure 7Ci). However, this superiority of 3DFect over Lipofectamine 3000 for cmRNA transfections of hDFs was not further observed in a 3D environment. Overall, Lipofectamine 3000 provided better support to pDNA than to cmRNA. The MFI revealed an enhancement in EGFP expression for pDNA when compared with cmRNA transfections, especially when Lipofectamine 3000 was used as vector (Figure 7Di, ii).

A higher number of EGFP-positive hOBs was obtained upon transfection with cmRNA complexes when compared with pDNA (Figure 7Ei, ii). Using cmRNA, the EGFP-positive hOBs reached values up to 29.4% ± 2.7% in monolayer transfection and up to 19.8% ± 4.2% for transfections performed using 3D scaffolds. Unexpectedly, MFI values were higher for Lipofectamine 3000 transfections with pDNA when compared with cmRNA (Figure 7Fi, ii). In fact, and remarkably, although Lipofectamine 3000 pDNA complexes transfected a moderate number of hMSCs and hOBs, they produced the highest amount of EGFP per cell as indicated by significantly higher MFI for most of the time points, both in monolayers (p < 0.0001, Figures 7Bi and 7Fi) and 3D environment (p < 0.0001, Figures 7Bii and 7Fii).

From the microscopic images taken after of EGFP transfection, it could be concluded that while cmRNA transfected cells are more ubiquitous (there is a higher number of EGFP-positive cells), they display less intense signals when compared with pDNA-transfected cells. Furthermore, EGFP-positive cells upon cmRNA transfection show a more homogeneous signal distribution whereas pDNA generates a rather random and heterogeneous EGFP-expressing populations. These facts are particularly noticeable for hMSCs and hDFs in Figures 8A and 8B. In hOBs; however, these differences could not be concluded from the microscopy images (Figure 8C).

### Transfection efficiency of cmRNA and pDNA upon delivery with a polymeric vector: Comparison with lipid Lipofectamine 3000

Contrasting differences on MetLuc activity were observed based on the use of a polymeric or a lipid vector. For all three cell types (i.e., hMSCs, hDFs, or hOBs) the use of cmRNA with the polymeric vector TransIT-X2 remarkably reduced protein expression when compared with the cmRNA lipoplexes (Figures S12A–S12C). Contrarily, this polymeric vector seemed to favor transfection of pDNA irrespective of the cell type. When hMSCs were transfected with TransIT-X2 pDNA complexes, protein expression was significantly higher compared with the pDNA lipid complexes, with an increased MetLuc activity over time (Figure S12 A). Similarly, in hOBs, the combination pDNA and TransIT-X2 significantly increased protein production compared with pDNA and Lipofectamine 3000 complexes for the first 3 days (Figure S12C). In hDFs, transfection efficiency with pDNA and TransIT-X2 only became significantly higher at day 5 and day 7 compared with complexes of pDNA and Lipofectamine 3000 (Figure S12B).

## Discussion

The field of regenerative medicine is based on the joint effort of multiple disciplines. One example is the combination of tissue engineering and gene therapy. The merge of these two disciplines seeks to develop biological tissue substitutes to transfer genetic material to cells to restore or improve tissue function. To guarantee the success of this approach, it is fundamental to understand the mechanisms that reign the cellular access to genetic material and the underlying gene transfer efficiencies.

The mode by which gene therapy vectors are internalized into cells quantitatively affects their uptake, intracellular trafficking, and, lastly, their gene expression. In our study, cellular uptake was predominantly mediated by caveolae followed by clathrin-mediated endocytosis in all three cell types, that is, hMSCs, hDFs, and hOBs. Some differences were observed that depended on the lipid used as a vector. Inhibition of the caveolae pathways showed a prevalent effect on transfection efficiency when analyzing protein expression profiles. Unlike cellular uptake, protein expression requires intracellular transport, release into the cytoplasm, and, in case of pDNA, nuclear import. Our results show that, while the nucleic acid complexes utilize diverse entry pathways, i.e., caveolae-mediated endocytosis and clathrin-dependent endocytosis, the latter was not effective in liberating the genetic material in the cytosol or transporting it to the nucleus. Cellular uptake results are in line with the endocytic pathways that could be predicted from the sizes of the complexes. It has been reported that particles with dimensions of ∼200 nm are typically internalized via clathrin-coated pits, while larger particles with a size ∼500 nm undergo caveolae-mediated endocytosis.^18^ Our nucleic acid complexes, which in most cases featured sizes >500 nm, appeared to be internalized by caveolae. However, it is important to keep in mind that internalization of lipid vectors is also largely influenced by other factors such as the vector shape, ratio of lipids to nucleic acids, and surface chemistry and charge.^19^^,^^20^ In fact, the latter not only affects cellular internalization but also impacts cytosolic release and subsequent protein production. Complexes having a net positive charge are usually perceived as better transfecting agents as they would be attracted to the negatively charged cell membrane. However, several research groups have shown that negatively charged vectors or neutral vectors, like the ones studied here, also successfully manage to transfer the encapsulated nucleic acids inside the cells and facilitate efficient transfection *in vitro* and *in vivo.*^21^^,^^22^^,^^23^ In fact, neutral and negatively charged nucleic acid complexes have been reported to be advantageous *in vivo* in that they prevent non-specific interactions between anionic plasma proteins and cationic lipoplexes, and its subsequent aggregation, deterioration, and/or removal from the circulation.^24^^,^^25^

Vesicle structures resembling caveolae appeared as flask-shaped invaginations in the plasma membrane of all cell types investigated. Less recurrently, clathrin-like vesicles were also found characterized by a more electron-dense membrane. These data are in agreement with the inhibition study results that revealed the dominant role of caveosome-mediated endocytosis in the uptake of the lipoplexes by all hMSCs, hDFs, and hOBs. Clathrin-mediated endocytosis has been acknowledged as the main pathway for internalization of extracellular components in most cell types.^26^ However, Pelkmans and Helenius, among other authors, acknowledged endocytosis via caveolae as a parallel uptake mechanism.^26^ They elegantly reported that caveolae transport their cargo to caveosomes, whereas clathrin-coated vesicles turn into early and late endosomes and finally lysosomes.^26^

In our study, lipoplexes internalized by clathrin-mediated endocytosis and other parallel mechanisms (e.g., phagocytosis in hObs) might have matured into endo-lysosomes that undergo enzymatic attack and degradation of their content. This observation was confirmed using specific drugs that either promoted or inhibited endosomal release. A temporal increase of protein production with the lysosomotropic agent chloroquine at early time points suggests that, while most complexes are able to release their cargo into the cytosol, part of the internalized complexes is naturally directed to late endosomes, which then mature into lysosomes. This might explain why high levels of uptake, for instance when hOBs were transfected with 3DFect, resulted in poor levels of protein expression. By using correlative light electron microscopy (CLEM), a colocalization of complexes with lysosome-like structures and with stained lysosomal compartments was appreciated, which is highly supportive of the previous findings. As expected, blocking endosomal release with bafilomycin caused accumulation of complexes in lysosomes and was detrimental to protein expression. Cervia et al. also reported a significant reduction in electro-transfection efficiency when COS7 and HEK293 cells were transfected after treatment with bafilomycin.^27^ On the basis of our data, we speculate that nucleic acid release from endosomes to the cytosol is not as effective as release from caveosomes.

One remarkable observation from our study was the differences in expression levels resulting from cmRNA or pDNA for each cell type investigated. We observed that, for fast dividing cells such as hDFs, pDNA yielded higher expression levels. Conversely, in slow-dividing hOBs, pDNA barely generated protein expression, while cmRNA was responsible for high protein production. It has been documented that the expression efficiency of a transgene is strongly dependent on the cell-cycle phase at transfection given that a non-virus is used.^28^ DNA insertion to the cell nucleus is not favored in slowly dividing cells.^29^ This is the case of osteoblasts, a cell type of high relevance in the field of bone regeneration. In this case, using cmRNA-based gene transfer may be highly beneficial. In the case of hMSCs, which present an intermediate doubling time compared with the other two cell types investigated here, the combination vector/nucleic acid seemed more relevant than the nucleic acid per se.

Along with lipid vectors, polymeric vectors have seen remarkable improvement in the last decade for gene therapy applications. Compared with lipoplexes, polymer-based delivery offers some advantages such as higher stability, chemical versatility, and ability to carry targeting moieties.^30^ In this study, striking difference emerged when comparing lipid and polymeric vectors. Overall, when using pDNA, the polymeric vector was more efficient, whereas cmRNA transfections increased when using lipid vectors. A possible explanation for this observation might be related to the differences in physical properties between the used vectors. Polymeric complexes almost doubled the size of the lipid vectors and were positively charged. While it is known that positively charged vectors have some advantages, such as better packing of the negatively charge nucleic acids, the advantage of such big complexes >1,000 nm are less obvious. Some authors describe that large complexes facilitate the formation of large intracellular vesicles during the entry pathway of endocytosis, which are easier to disrupt and may cause a more effective nucleic acid release into the cytosol.^31^ Altogether, this indicates the importance on the selection of the vectors that could favor transfection depending on the nucleic acid used.

The cellular microenvironment is also relevant to nucleic acid uptake and subsequent protein translation. In this study, we used 3D nanofibrous scaffolds with fibers closely mimicking the physical dimensions and fibrillar structure of the extracellular matrix.^32^^,^^33^ This ECM resemblance, together with the hydrophilic character of the scaffolds, may be responsible for the increased protein expression levels observed when compared with 2D, monolayer transfection. This was particularly clear when hMSCs and hDFs were used. Previous studies have also shown enhanced transfection in hydrophilic surfaces,^34^ probably explained by the improved cell attachment, spreading, and cytoskeletal organization of these surfaces.^35^ Our previous studies also demonstrated the suitability of 3D fibrin gels^36^^,^^37^ and MBCP ceramic particles^36^ as carriers for cmRNA molecules. Both biomaterials resulted in superior gene expression when compared with 2D cell monolayer transfections. We explained this result by the superior cell viability observed in the 3D matrices after transfection.^36^ These results are in line with the observations of Badieyan et al. who showed higher, prolonged protein expression in MSCs transfected with MetLuc cmRNA loaded in collagen sponges when compared with 2D transfections.^38^ In 2D, the authors reported a rapidly fading protein expression. This evidence indicates the crucial role of 3D matrices in cmRNA transfer to the cells.

A distinct difference observed in our study when comparing cmRNA with pDNA transfections was that cmRNA showed a rather early onset of expression compared with the delayed peaks obtained when pDNA was used. This behavior was generic and similar time distributions were observed for hMSCs, hDFs, and hOBs. This observation could be explained based on the cell-division process. While pDNA complexes need to wait for cells to enter mitosis for reaching the nucleus, cmRNA molecules released from cmRNA lipoplexes are translated immediately after cytosolic release. Consequently, cmRNA-induced protein expression starts to occur earlier than the pDNA-mediated one. Interestingly, transfection of hMSCs with cmRNA induced a second peak of expression typically at day 3. This could indicate that cmRNA expression is also somehow linked to cell cycle, or, as described by Raj et al., such kinetics could be due to the stochasticity of mRNA known to produce intrinsically random “transcription bursts.”^39^ Our EGFP expression studies highlighted further differences between cmRNA and pDNA. In our study, transfection with pDNA produced more copies of the desired protein but typically reaching less cells. This was particularly clear for hMSC and hOB transfections. Similar observations were reported by Leonhardt et al., who described that cells transfected with mRNA have an earlier and ubiquitous onset of expression compared with the delayed onsets of pDNA-transfected cells using human alveolar adenocarcinoma cells (A549), HeLa cells, and Madin-Darby canine kidney epithelial cells (MDCKII).^40^ Apart from the inability of pDNA to transfect non-dividing or slow-dividing cells, a further disadvantage of pDNA transfection was the generation of cytotoxicity. This was observed as a reduction in cellular metabolic activity when compared with cells transfected with cmRNA vectors. A possible explanation could be related to the fact that DNA presence in the cytoplasm is not normal for eukaryotic cells. Once in the cytoplasmic space, the foreign DNA might be detected by molecules, such as the Toll-like receptors (TLR), which may polarize cells toward pro-inflammatory, antigen-presenting-like and apoptotic phenotypes.^41^ We must keep in mind that foreign mRNA also has the capacity to activate TLR. However, the modifications in the structure of the cmRNA are precisely designed to reduce the detection of mRNA by these molecules. One example is the replacement of the nucleotides like uridine, which is recognized by TLR7, with modified nucleotides such as pseudouridine, 2-thiouridine, or 5-methyluridine which are undetected by the same receptors.^42^

In conclusion, a summary of our main findings is presented as follows.

(1) Clathrin- and caveolae-mediated pathways are both equally important routes of uptake of lipoplexes. However, caveolae-mediated endocytosis favors endosomal escape of the complexes and therefore is the most productive route for gene delivery. This holds true for both cmRNA and pDNA.
(2) cmRNA was shown to effectively transfect osteoblasts, which are slow- and/or non-dividing cells compared with pDNA, which was more efficient in fast-dividing fibroblasts.
(3) Complexation with lipid vectors favored delivery of cmRNA, while polymeric vectors improved delivery of pDNA.
(4) cmRNA exhibited earlier boosts of protein production compared with pDNA. In either case, higher transfection efficiencies were observed when cells grew in a 3D scaffold, highlighting the relevance of studying nucleic acid transfer in a setting that mimics the native environment of cells.
(5) Transfection with cmRNA reduced cytotoxicity compared with pDNA transfection.

In addition to the above highlighted findings, the low manufacturing costs and safety of cmRNA compared with pDNA makes cmRNA an excellent therapeutic candidate for the applications described. This study provides valuable insights into the rationale behind the choice of nucleic acids and their carriers for gene delivery.

## Materials and methods

### Nucleic acids

Two different nucleic acids were used in this study, pDNA and chemically modified mRNA. In both cases, the nucleic acids encoded either MetLuc or EGFP. MetLuc pDNA and EGFP pDNA were both produced by PlasmidFactory (Bielefeld, Germany). MetLuc cmRNA and EGFP cmRNA were designed in-house, synthesized via T7 RNA polymerase *in vitro* transcription, and purified following our previously reported protocol.^43^^,^^44^ The sequences of all nucleic acids used in this study can be found in the Dataverse repository (https://dataverse.nl/privateurl.xhtml?token=45ace5b3-fba3-48c6-b264-0513ac98398c).

### Fluorescent labeling of MetLuc coding nucleic acids

For later investigations of cellular uptake, internalization, and trafficking of the nucleic acid-lipid complexes, MetLuc-coding nucleic acids were fluorescently labeled using the Label IT MFP488 Labeling Kit (Mirus Bio, Madison, WI). The labeling reagent contains a fluorophore (i.e., MFP488) linked to a reactive alkylating group that covalently attaches to any reactive heteroatom within the nucleic acids. The resulting MFP488-MetLuc cmRNA was purified by ammonium acetate (Sigma-Aldrich, St. Louis, MO) precipitation and washed with ethanol 70%. MFP488-MetLuc pDNA was purified by precipitation with 5M sodium chloride (Sigma-Aldrich) and ice-cold 100% ethanol. The labeling density (pmol of dye/μg of nucleic acid) and the base-to-dye ratio were calculated as per the manufacturer’s instructions (Table S3). MFP488-MetLuc pDNA or MFP488-MetLuc cmRNA were used only when specified.

### Formation of transfection complexes

Complexes were always freshly prepared using non-supplemented Opti-MEM (Life Technologies, Carlsbad, CA) by mixing selected lipid vectors (Lipofectamine 3000, Invitrogen, Waltham, MA) or 3DFect (OzBiosciences, Marseille, France) with respective nucleic acids (pDNA or cmRNA). The nucleic acid to vector ratio was 1:2, and the volume to weight ratios were followed as described in the instructions provided by the vector manufacturers. Similarly, polymer-based complexes were prepared by mixing TransIT-X2 (Mirus Bio, Madison, WI) with either pDNA or cmRNA in non-supplemented Opti-MEM with a nucleic acid to vector ratio of 1:3. The nucleic acid-vector mixtures were incubated for 20 min at room temperature to allow complex assembling and formation.

### Characterization of complexes

The size, PdI, and zeta potential of the nucleic acid-lipid or nucleic acid-polymer complexes were investigated using dynamic light scattering methods using a Zetasizer Nano ZSP (Malvern Instruments, Worcestershire, UK). In brief, 800 μL of the nucleic acid-vector complex solution (formed in non-supplemented cell culture medium) was used for the measurements, which were carried out at a fixed angle (173° backscattering). Indicated cuvettes for electrophoretic measurements (DTS1070, Malvern Instruments) were used. All the samples were measured at 20°C ± 3°C and the measurements were performed in triplicate (n = 3).

The morphology of the lipid complexes was examined using TEM. In brief, a 5 μL solution of each lipid complex was loaded onto a 300-mesh copper grid coated with a carbon support film. The grids were then air-dried overnight and examined with a TEM (Tecnai G2 Spirit BioTWIN iCorr, FEI, Hillsboro, OR) operated at 120 kV. Imaging was carried out using a WA-Veleta camera (EMSIS, Münster, Germany).

### Encapsulation efficiency of the nucleic acids by the lipid carriers

The encapsulation efficiency was assessed for each lipid complex. PicoGreen dsDNA (Molecular Probes, Eugene, OR) or QuantiFluor RNA (Promega, Madison, WI) were used for pDNA- or cmRNA-based complexes, respectively. In brief, standard curves of a known concentration of DNA or RNA were prepared as per the manufacturer’s instructions. In parallel, 1 mL of the complexes were prepared. Then, 10 μL of the complexes or standards were added to 96-well plates containing the nucleic acid-binding dye, mixed thoroughly, and incubated for 5 min at room temperature protected from light. Fluorescence was measured (excitation at 492 nm and emission at 540 nm) using a CLARIOSTAR plate reader (BMG Labtech, Ortenberg, Germany). The percentage of encapsulated nucleic acids was calculated using the following equation.

### Cells and cell culture

Three different primary cells of human origin were used in the study, namely hMSCs, hDFs, and hOBs. hMSCs cells were isolated with written informed donor consent from the iliac crest (male, age 17, Maastricht University Medical Center, Maastricht, the Netherlands) following ethical approval from the local and national authorities. All procedures were carried out in accordance with the declaration of Helsinki in its latest amendment. hMSCs were isolated and sub-cultured as described previously.^45^ In brief, aspirates were resuspended using a 20-gauge needle, and nucleated cells were plated at a density of 500,000 cells/cm^2^. The cells obtained from the first trypsinization were considered as passage 1. Cells were characterized by flow cytometry and their ability to proliferate and differentiate as described before.^45^ hMSCs were cultured in α-minimal essential medium (Life Technologies) with GlutaMAX supplemented with 10% heat-inactivated fetal bovine serum (FBS) (Sigma-Aldrich) and 1% penicillin/streptomycin (PS) (100 U/mL, Thermo Fisher Scientific, Waltham, MA).

hDFs obtained from neonatal dermis were purchased from Lonza (Basel, Switzerland) and grown, as recommended by the manufacturer, in Dulbecco’s modified Eagle medium (DMEM) (Thermo Fisher Scientific) supplemented with 10% FBS and 1% PS.

Similarly, hOBs obtained from humerus spongy bone tissue, were purchased from Lonza and cultured in DMEM/Nutrient Mixture F-12 1:1 (Thermo Fisher Scientific) with GlutaMAX and supplemented with 10% FBS, 1% PS, and 50 μg/mL ascorbic acid.

Cell culture for all used cell types was performed in a humidified atmosphere at 5% CO_2_ level at 37°C.

### Nucleic acid transfection in cell monolayers

Cells (i.e., hMSCs, hDFs, or hOBs) were seeded as described in Table 1 using a standard monolayer cultivation (2D) method and a cell suspension containing 50,000 cells/mL. After 24 h, cells were transfected with either cmRNA or pDNA complexes formed previously with either Lipofectamine 3000, 3DFect, or TransIT-X2. The formation of transfection complexes has been described above. After 6 h of incubation with the complexes, the medium was removed and fresh Opti-MEM containing 10% FBS and 1% PS was added. Depending on further assays to be performed, different plate formats, volumes, and nucleic acid-lipid or nucleic acid-polymeric complexes were used. This information is summarized in Table 1.

**Table 1:** Summary of the transfection conditions used for cell monolayers and cells seeded on scaffolds with pDNA and cmRNA using the lipid vectors Lipofectamine 3000, 3DFect, or TransIT-X2

Transfection in cell monolayers (2D)					
Assays	Plate format	Volume per well (μL)	Nucleic acid	Lipid	Nucleic acid:lipid ratio
Endocytosis pathway determination	24-well plate	500	MFP488-MetLuc pDNA MFP488-MetLuc cmRNA	3DFect Lipofectamine 3000	1:2
				3DFect Lipofectamine 3000	
Endocytosis pathway determination, MetLuc expression, and cell viability	96-well plate	100	MetLuc pDNA MetLuc cmRNA	3DFect Lipofectamine 3000	1:2
				3DFect Lipofectamine 3000	
CLEM (cellular uptake visualization of complexes)	Ibidi μ-Slide 8-well	300	MFP488-MetLuc pDNA MFP488-MetLuc cmRNA	3DFect Lipofectamine 3000	1:2
				3DFect Lipofectamine 3000	
Microscopy and flow cytometry of EGFP-positive cells	24-well plate	500	EGFP pDNA EGFP cmRNA	3DFect Lipofectamine 3000	1:2
				3DFect Lipofectamine 3000	
Trafficking of internalized complexes	96-well plate	100	MFP488-MetLuc	Lipofectamine 3000 TransIT-X2	1:2 1:3 1:2 1:2 1:3
			MFP488-MetLuc MetLuc cmRNA	Lipofectamine 3000 3DFect TransIT-X2	

**Transfection of cells on scaffolds (3D)**					

MetLuc expression and cell viability	96-well plate	100	MetLuc pDNA MetLuc cmRNA	3DFect Lipofectamine 3000	1:2
				3DFect Lipofectamine 3000	
Microscopy and flow cytometry of EGFP-positive cells	24-well plate	500	EGFP pDNA EGFP cmRNA	3DFect Lipofectamine 3000	1:2
				3DFect Lipofectamine 3000	

The transfection conditions were adjusted to the specific assay to be performed.

### Endocytosis pathway determination of lipoplexes

The mechanism of cellular uptake of the different complexes was studied through inhibition of specific endocytic pathways. Therefore, the inhibitors chlorpromazine, wortmannin, and genistein were selected to block the clathrin-mediated endocytosis, macropinocytosis, and caveolae-mediated endocytosis, respectively. Table S4 summarizes the endocytic route and mechanism of action of each inhibitor selected.^46^^,^^47^^,^^48^ All inhibitors were purchased from Sigma-Aldrich.

*In vitro* toxicity of chlorpromazine, wortmannin, and genistein is known to be dose dependent.^17^ Therefore, the cytotoxicity of these reagents was evaluated using PrestoBlue Cell Viability assay (Invitrogen). hMSCs, hDFs, or hOBs were seeded in 96-well plates at a density of 15,000 cells/cm^2^. After 24 h, cells were incubated in Opti-MEM supplemented with 10% FBS and 1% PS and various concentrations of the described inhibitors for 2 h in a cell culture incubator at 37°C and 5% CO_2_. The concentration range evaluated for each of the inhibitors used is shown in Table S5. Subsequently, the cells were incubated with the PrestoBlue reagent (10 μL substrate and 90 μL non-supplemented Opti-MEM per well). After 1 h under standard cell culture conditions and protection from direct light, the supernatants were collected for measurement in a new 96-well plate. Fluorescence was measured on a CLARIOSTAR plate reader (BMG Labtech) at an excitation wavelength of 535 nm and an emission wavelength of 615 nm. Normalization of the obtained data was performed using the values of the untreated cells. Results are reported as a percentage of cell metabolic activity.

The endocytic pathways employed by the cells to uptake the different nucleic acid-lipid complexes were investigated using two approaches, that is to (1) quantify complexes internalized via specific endocytic routes and (2) quantify protein production after transfection post-blockade of specific endocytic routes. For both studies, chlorpromazine, wortmannin, and genistein were used to block specific endocytic pathways before and during transfection.

Flow cytometry was used to quantify complexes internalized via specific endocytic routes. In brief, cells (i.e., hMSCs, hDFs, and hOBs) were seeded in 24-well plates at a cell density of 15,000 cells/cm^2^. After 24 h of cell seeding and prior to transfection, cells were pre-incubated with different inhibitors at the previously selected concentrations (Table S5) in non-supplemented Opti-MEM for 1 h. Thereafter, cells were incubated with the different, freshly prepared nucleic acid complex solutions for a further 1 h. Transfection complexes containing MFP488-MetLuc pDNA or MFP488-MetLuc cmRNA were prepared with either Lipofectamine 3000 or 3DFect as described previously in formation of transfection complexes. Next, complexes were added to a non-supplemented Opti-MEM solution containing the specific inhibitor at the selected concentration. After the desired incubation period was completed, the medium containing nucleic acid-lipid complexes and inhibitors was removed and the cells were rinsed three times with PBS (Gibco, Waltham, MA). Thereafter, cell monolayers were prepared for flow cytometric analysis. In brief, cells were detached with 0.05% trypsin (Gibco), centrifuged at 500 × *g* for 5 min, and resuspended with ice-cold PBS. The uptake of the fluorescent nucleic acid-lipid complexes was analyzed using a BD Accuri C6 Plus Flow Cytometer (BD Biosciences, Franklin Lakes, NJ). Forward and side scatter density plots were used to identify the cell population and 5,000 events were collected per sample. FlowJo Software v.10 (BD, Franklin Lakes, NJ) was used for the analysis. Cellular uptake of the labeled complexes was calculated as the percentage MFP488-positive cells in the 533/30 filter relative to untransfected cells (absence of MFP488 signal). A total of n = 3 samples were used per group for the measurements.

Further investigations on the endocytic internalization mechanisms were performed by quantifying the protein production after transfection post-blockade of specific endocytic routes. For this, cells were pre-incubated with the inhibitors as described previously. Next, transfections were performed with MetLuc pDNA or MetLuc cmRNA with either Lipofectamine 3000 or 3DFect in the presence of the specific inhibitors. After 6 h, the medium containing nucleic acid-lipid complexes and inhibitors was replaced by Opti-MEM supplemented with 10% FBS and 1% PS. Transfections were performed as described in nucleic acid transfection in cell monolayers, with transfection parameters summarized in Table 1. MetLuc activity over time was quantified up to 7 days following the protocol described hereafter. A total of n = 5 samples were used per group for the measurements.

### Cellular internalization of lipoplexes

To study the internalization of the nucleic acid-lipid complexes, CLEM was used. This technique couples confocal microscopy with TEM images allowing detailed visualization of internal subcellular structures while locating fluorescent-labeled molecules. In brief, cells (i.e., hMSCs, hDFs, and hOBs) were seeded on μ-Slide 8-well (Ibidi, Gräfelfing, Germany) culture dishes at a density of 15,000 cells/cm^2^. The Ibidi μ-Slide features an imprinted coordinate system that allowed the precise localization of individual and specific cells in the TEM after the desired ROI was imaged with confocal microscopy (Figure S13). After 24 h, cells were transfected with MFP488-MetLuc pDNA or MFP488-MetLuc cmRNA (using either Lipofectamine 3000 or 3DFect, Table 1). After 1 h of incubation, cells were washed 3 times with PBS and fixed with 1.6% glutaraldehyde (Merck, Gernsheim, Germany) for 20 min at room temperature. Samples were washed twice with PBS, cell nuclei were stained with Hoechst 34580 (Thermo Fisher Scientific) and actin filaments were stained with phalloidin Alexa Fluor (AF647, Thermo Fisher Scientific). Samples were kept in PBS for imagining purposes. Overview pictures of the coordinate system were acquired using a 10× dry objective (HC PL FLUOTAR 10×/0.30 DRY, Leica Microsystems, Wetzlar, Germany) on an inverted SP8 confocal microscope (Leica Microsystems). High-resolution images of the ROI were acquired with the same microscope using an 86× water immersion objective (HC PL APO 86×/1.20 WATER, Leica Microsystems). A White Light Laser was tuned to 488 nm (detects MFP488) and 647 nm (detects AF647), whereas a separate 405 nm laser was used for the Hoechst channel. Confocal images were acquired in the middle of the coordinate system of each well, and tiles of 1,024 × 1,024 pixels were acquired and stitched using the LasX software (Leica Microsystems).

The same samples were processed by TEM. For this, glutaraldehyde-fixed samples were kept in 0.1 M cacodylic acid sodium salt trihydrate (cacodylate buffer; Thermo Fisher Scientific) at 4°C for 24 h. Subsequently, samples were washed (thrice, 15 min each) with 0.1 M cacodylate buffer and incubated in a buffer containing 1% osmium tetroxide (Electron Microscopy Sciences, Hatfield, PA) and 1.5% potassium ferricyanide (K_3_[Fe(CN)_6_], Merck) protected from the light at 4°C for 1 h. Then, the samples were dehydrated in a graded series of ethanol (70%–90%–100%, Merck), with each step repeated twice for 30 min. Subsequently, the samples were infiltrated with Epon resin (LADD Research, Williston, VT) for 2 days, embedded in the same resin and polymerized at 60°C for 2 days. The Epon blocks were trimmed down and sectioned using a UC6 ultramicrotome (Leica Microsystems) and a diamond trimming knife (Diatome, Hatfield, PA). Selected cells within the ROI were imaged using a TEM (Tecnai G2 Spirit BioTWIN iCorr, FEI) operated at 120 kV and using a WA-Veleta camera (EMSIS). The acquired TEM images of each cell were overlapped with the confocal images of the same cell. Specific areas within the cell where MFP488-MetLuc nucleic acid-lipid complexes were located were further inspected at higher magnifications and imaged. Images were processed to enhance contrast on Fiji software (https://fiji.sc/).^49^ Superimposition and correlation of confocal and TEM photomicrographs was done manually in Adobe Illustrator (CC 2018, San José, CA). The imprinted coordinated system and nuclei patterns allowed an easy recognition of the ROI. Opacity of confocal images was reduced to 40% and then images were superimposed to the corresponding TEM images.

### Endosomal and lysosomal trafficking of internalized complexes

Cells (i.e., hMSCs, hDFs, or hOBs) were seeded onto 96-well plates using a cell suspension of 50,000 cells/mL. After 24 h, cells were treated with the endosome acidification-interfering drugs chloroquine at 25 μM (Sigma-Aldrich) or bafilomycin A1 at 200 nM (Sigma-Aldrich). Transfections were performed at 4 h (chloroquine) or 2 h (bafilomycin) post-treatment using either MetLuc cmRNA or pDNA complexes formed previously with either Lipofectamine 3000 or TransIT-X2. After 6 h incubation with the nucleic acid complexes in the presence of the drugs, the medium was removed and fresh Opti-MEM with 10% FBS and 1% PS was added. Supernatants were collected at days 1, 2, 3, 5, and 7 after transfection, and MetLuc activity was quantified following the protocol described hereafter. A total of n = 4 samples were used per group for the measurements.

To visualize a possible colocalization of internalized complexes with lysosomes, LysoTracker Deep Red (150 nM, Invitrogen) was used in addition to chloroquine or bafilomycin. Transfection complexes containing MFP488-MetLuc cmRNA were prepared with either Lipofectamine 3000, 3DFect, or TransIT-X2 as described previously. After 3 h incubation with complexes, cells were washed with PBS, fixed with 4% paraformaldehyde (Sigma-Aldrich), and stained with Hoechst 34580 (Thermo Fisher Scientific). High-resolution images were acquired with the same microscope as described above using a 100× oil immersion objective (HC PL APO 100×/1.20 OIL, Leica Microsystems). A time-lapse video was performed on an automated inverted Nikon Ti-E microscope (Nikon Eclipse Ti-E, Nikon Europe, Amsterdam, the Netherlands) equipped with a Lumencor Spectra X light source, a Photometrics Prime 95B sCMOS camera (Photometrics, Krailling, Germany), an MCL NANO Z500-N TI z-stage, and a Okolab incubator (37°C, 5% CO_2_) for live cell imaging (Okolab, Pozzuoli, Italy). Acquisition took place using a 40× objective.

### Fabrication and characterization of ES scaffolds

To study transfection in a 3D environment, ES scaffolds were fabricated using a block copolymer, poly(ethylene oxide terephthalate)/poly(butylene terephthalate) (PEOT/PBT). PEOT/PBT-based electrospun meshes have recently emerged as a promising nanofibrous substrate for diverse tissue engineering applications.^50^^,^^51^ A previously reported, an electrospinning setup was implemented for the fabrication process.^33^^,^^52^ The 300PEOT55PBT45 grade of PEOT/PBT copolymer (kindly provided by PolyVation, Groningen, the Netherlands) was used, where 300 represents the molecular weight (g/mol) of the initial polyethylene glycol used in the copolymerization reaction, and 55/45 denotes the weight ratio of PEOT and PBT, respectively. The precursor polymeric solution was prepared by dissolving 17% (w/v) PEOT/PBT in a 70:30 (v/v) solvent mixture of trichloromethane (anhydrous, Sigma-Aldrich) and hexafluoro-2-propanol (analytical reagent grade, Biosolve, Valkenswaard, the Netherlands), respectively. The dissolution was performed under sealed environment with overnight stirring at ambient conditions.

A Fluidnatek LE-100 electrospinning system (Bioinicia, Valencia, Spain) was used in a controlled environment, where the chamber temperature was set at 23°C and relative humidity at 40%. The PEOT/PBT precursor solution was discharged through a 0.8 mm needle at a flow rate of 0.9 mL/h. A cylindrical mandrel (diameter = 200 mm, length = 300 mm) rotating at 200 revolutions/minute was used as the collector. Aluminum foil assembled with supporting bands (inner diameter = 12 mm, outer diameter = 15 mm) of Finishmat 6691 LL (Lantor, Veenendaal, the Netherlands) was wrapped on the rotating mandrel to collect the electrospun nanofibers. Finally, a voltage potential difference of 12.5 kV was applied between the needle (10 kV) and the collector (−2.5 kV) with the working distance maintained at 10 cm. The electrospinning was performed for a duration of 30 min to manufacture nanofibrous meshes with the desired morphology.

SEM was performed to examine the nanofiber morphology (top view) and thickness (cross-sectional view) of the electrospun meshes. A thin layer of gold coating was applied (SC7620 sputter coater, Quorum Technologies, Lewes, UK) over the samples before imaging them on the SEM (JSM-IT200, JEOL, Tokyo, Japan). The images were captured at 3,000× magnification, at an accelerating voltage of 10 kV and working distance of 10 mm. The subsequent image analysis was performed on Fiji software (https://fiji.sc/)^49^ to determine fiber diameter and thickness of the scaffolds.

The hydrophilicity of both substrates, the ES scaffolds (3D), and the polystyrene culture plates (2D), was tested using a drop shape analyzer (DSA25S, Krüss, Hamburg, Germany). A sessile water droplet of 4 μL was deposited on each sample, following which its contact angle was calculated based on the Laplace-Young computational method. The measurement was performed for an extended duration of time considering the dispersal of the water droplet.

### Nucleic acid transfection of cells seeded on the ES scaffolds

Circular scaffolds of 6 and 15 mm were punched to fit into 96- and 24-well plates, respectively. Punched scaffolds were placed inside the wells and were held by sterile O-rings (ERIKS, Alkmaar, the Netherlands). Scaffolds were then disinfected with ethanol for 60 min, followed by a drying step inside the biosafety cabinet for 3 h to let the ethanol evaporate. Next, scaffolds were washed twice with PBS and incubated overnight in cell culture medium. Cells (i.e., hMSCs, hDFs, or hOBs) were seeded onto the ES scaffolds using a cell suspension of 50,000 cells/mL and the volumes specified in Table 1. After 24 h, cells were transfected with either cmRNA or pDNA lipid complexes formed previously with either Lipofectamine 3000 or 3DFect, as described in section formation of transfection complexes. After 6 h of incubation with the complexes, the medium was removed and fresh Opti-MEM with 10% FBS and 1% PS was added. A summary of the transfection parameters used is given in Table 1.

### Transfection efficiency of nucleic acids, cmRNA, and pDNA, using either 2D or 3D cell culture environment

#### MetLuc activity

Supernatants used to evaluate MetLuc activity were collected from the cell monolayer (2D) or cell-seeded scaffold (3D) on days 1, 2, 3, 5, and 7 after transfections were performed. Supernatants were stored at −80°C until further evaluation of MetLuc expression. To quantify MetLuc expression, 50 μL of native coelenterazine (50 μM in degassed sodium phosphate buffer [pH 7.0]; Synchem, Felsberg, Germany) was added to 50 μL of the supernatant in a white opaque 96-well plate. Luminescence intensity was measured instantly at 480 nm and reported as RLU at room temperature using a CLARIOSTAR plate reader (BMG Labtech). A total of n = 5 samples were used per group for the measurements.

#### Evaluation of EGFP-positive cells and fluorescence intensity

Fluorescence images of the cell monolayer (2D) or cell-seeded scaffold (3D) were acquired on days 1, 2, 3, 5, and 7 after transfections with EGFP cmRNA or EGFP pDNA lipid complexes. Imaging was performed on the same automated inverted Nikon Ti-E microscope as described before using 10× and 40× objectives to visualize transfection differences between conditions and monitor morphological changes.

The percentage of EGFP-positive cells was quantitatively assessed by flow cytometry. For this, after each time of observation was reached, cells were washed with PBS, trypsinized, and transferred to microtubes. Detachment with trypsin was adjusted to a duration of 10 min for cell-seeded scaffolds (3D) and to 5 min for cell monolayers (2D). Next, cells were centrifuged at 500 × *g* for 5 min and resuspended in ice-cold PBS for flow cytometric analysis. The percentage of EGFP-positive cells and the MFI was analyzed using a BD Accuri C6 Plus Flow Cytometer (BD Biosciences) equipped with a 488 nm argon laser for excitation. For each sample, 5,000 events within the cell population were gated based on forward and side scattering parameters. The percentage of EGFP-positive cells and the MFI were determined relative to the untransfected cell population using FlowJo Software v.10 (BD).

#### Cell viability after transfection

PrestoBlue cell viability assay was performed following the previously described procedure. Time of observation included days 1, 2, 3, 5, and 7 post-transfection. Cells were incubated with the PrestoBlue reagent for 1 h, after which the supernatants were collected to measure fluorescence. Results were reported as a percentage of cell metabolic activity of transfected cells relative to untransfected cells. Samples were measured in triplicate (n = 3).

### Comparison of transfection efficiency of lipid and polymeric vectors

Cells (i.e., hMSCs, hDFs, or hOBs) were seeded onto 96-well plates and using a cell suspension of 50,000 cells/mL. After 24 h, cells were transfected with either MetLuc cmRNA or pDNA complexes formed previously with either Lipofectamine 3000 or TransIT-X2. Instructions provided by the manufacturer of either transfection reagent were followed exactly. Supernatants were collected and MetLuc activity was quantified as described above. A total of n = 4 samples were used per group for the measurements.

### Statistical analysis

All the obtained values are reported as mean ± standard deviation. Statistical analysis was performed using GraphPad Prism v.8.00 (GraphPad Software, San Diego, CA). Gaussian distribution of the data was verified by D’Agostino-Pearson test. One- or two-way ANOVA followed by a multiple comparisons test was performed to analyze multiple groups as follows; one-way ANOVA with post hoc Tukey was used to analyze the encapsulation efficiency and concentration-dependent toxicity of the endocytosis inhibitors. Similarly, two-way ANOVA also corrected by Tukey was applied to the eGFP expression and cell metabolic activity data. Furthermore, two-way ANOVA followed by Sidak’s multiple comparisons test was performed to analyze the size and zeta potential data as well as the MetLuc activity. Finally, the cellular uptake data were analyzed by two-way ANOVA followed by Dunnett’s test. The exact methodology followed for statistical analysis has been described in the legend of each figure, along with the sample size (n) used in each case. Probabilities of p < 0.05 were considered significant. p values are reported as ∗p ≤ 0.05, ∗∗p ≤ 0.01, ∗∗∗p ≤ 0.001, and ∗∗∗∗p ≤ 0.0001. AUC values were calculated using GraphPad Prism.

## Data availability

Data generated or analyzed during this study are presented. Structures and sequences of the cmRNAs and the plasmids are available in the Dataverse repository (https://dataverse.nl/privateurl.xhtml?token=45ace5b3-fba3-48c6-b264-0513ac98398c).

## Supplemental information

Document S1. Figures S1–S13 and Tables S1–S5

Document S2. Article plus supplemental information
