# Supplementary material for: Cellular uptake of modified mRNA occurs via caveolae-mediated endocytosis, yielding high protein expression in slow-dividing cells
Source: Mol Ther Nucleic Acids. 2023 May 20;32:960–79. doi: 10.1016/j.omtn.2023.05.019 (PMC10250585; doi:10.1016/j.omtn.2023.05.019)
Supplement: Document S1. Figures S1–S13 and Tables S1–S5 [file mmc1.pdf]

OMTN, Volume *32*

Supplemental information

Cellular uptake of modified mRNA occurs via caveolae-mediated endocytosis, yielding high protein expression in slow-dividing cells

Claudia Del Toro Runzer, Shivesh Anand, Carlos Mota, Lorenzo Moroni, Christian Plank, Martijn van Griensven, and Elizabeth R. Balmayor

Table S1. Dynamic and electrophoretic light scattering characterization of cmRNA and pDNA complexes. Complexes were formulated at a nucleic acid: vector ratio (w/w) of 1:2 and 1:3 for lipid and polymeric complexes, respectively. Each value represents the mean ± SD.

Gene	Complex	Mean hydrated diameter (nm)	Polydispersity index, PdI	Electrokinetic potential (mV)
Metridia Luciferase	Lipo + cmRNA	436 ± 72	0.30	-4 ± 1.1
	Lipo + pDNA	709 ± 21	0.30	-0.1 ± 0.9
	3DFect + cmRNA	815 ± 78	0.29	-20 ± 0.3
	3DFect + pDNA	569 ± 99	0.30	-20 ± 2.6
	TransIT-X2 + cmRNA	1510 ± 47	0.43	16 ± 3.7
	TransIT-X2 + pDNA	1760 ± 245	0.41	4.2 ± 0.1
EGFP	Lipo + cmRNA	500 ± 90	0.30	-0.04 ± 1.2
	Lipo + pDNA	496 ± 126	0.29	0.43 ± 1.2
	3DFect + cmRNA	633 ± 121	0.38	-2.64 ± 4.8
	3DFect + pDNA	202 ± 12	0.21	-29 ± 1.5

Table S2. Cellular growth parameters. Growth constant and doubling time for each primary cells calculated with the exponential cell growth equation.

Cell type	Initial cell no.	Days in culture	Final cell no.	Growth constant	Doubling time
**hMSCs**	1.00×10^6	6	7.59×10^6	0.34	2.05
**hDFs**	1.11×10^6	4	13.5×10^6	0.62	1.11
**hOBs**	0.55×10^6	13	1.41×10^6	0.0720	9.63

Table S3. Labelling density of MFP488-MetLuc nucleic acids.

Sample	A_{260}	A_{dye}	$C.F._{260}$	ε_{dye}	ε_{base}	A_{base}	Base: Dye ratio	pmol dye / µg nucleic acid
Labelled MetLuc cmRNA	0.126	0.002	0.1	90,000	8,250	0.1	545.4	44.09
Labelled MetLuc pDNA	0.09	0.002	0.1	90,000	6,600	0.09	613.6	49.38

$\mathbf{A_{260}}$**:** Spectrophotometer readings (at 260 nm)

$\mathbf{A_{dye}}$**:** Maximum absorbance wavelength for MFP400 (A_{501})

$\mathbf{C.F._{260}}$**:** Constant value determined by dividing the absorbance of the free dye at 260 nm with that at A_{dye}

$\boldsymbol{\varepsilon_{dye}}$**:** Extinction coefficient of nucleic acid bound dye $\boldsymbol{\varepsilon_{dye}} = \frac{1}{(M \times cm)}$

$\boldsymbol{\varepsilon_{base}}$**:** Extinction coefficient of nucleic acid ($M^{-1}cm^{-1}$) $\boldsymbol{\varepsilon_{base}} = \frac{1}{(M \times cm)}$

Table S4. Summary of the endocytic pathways studied, their chemical inhibitors, and mechanisms of action.

Endocytic route	Chemical inhibitor	Mechanism of action
Clathrin mediated endocytosis	Chlorpromazine	Translocates clathrin and its adapter protein AP-2 from the plasma membrane to the late endosomal compartment, thus inhibiting clathrin-coated pit formation.[1]
Marcopinocytosis	Wortmannin	Phosphatidylinositol 3-kinase inhibitor, which disrupts the formation of macropinosomes and early endosome fusion.[2]
Caveolae mediated endocytosis	Genistein	Tyrosine-kinase inhibitor that causes local disruption of the actin network at the site of endocytosis and inhibits the recruitment of dynamin II, both indispensable events in the caveolae-mediated uptake mechanism.[3]

Table S5. Range of concentration tested for each inhibitor to assess cytotoxicity. Experiments were performed using hMSCs, hDFs, and hOBs. Based on the cytotoxicity evaluation, a concentration was selected for each inhibitor to be used in further experiments. The selected concentration is indicated in bold and underlined for each compound.

**Chlorpromazine (µM)**	**Wortmannin (µM)**	**Genistein (µM)**
5.6	0.01	100
11.3	0.02	150
16.9	0.03	**<u>200</u>**
22.5	0.04	250
**<u>28.1</u>**	**<u>0.05</u>**	300
33.8	0.06	350
39.4	0.07	400

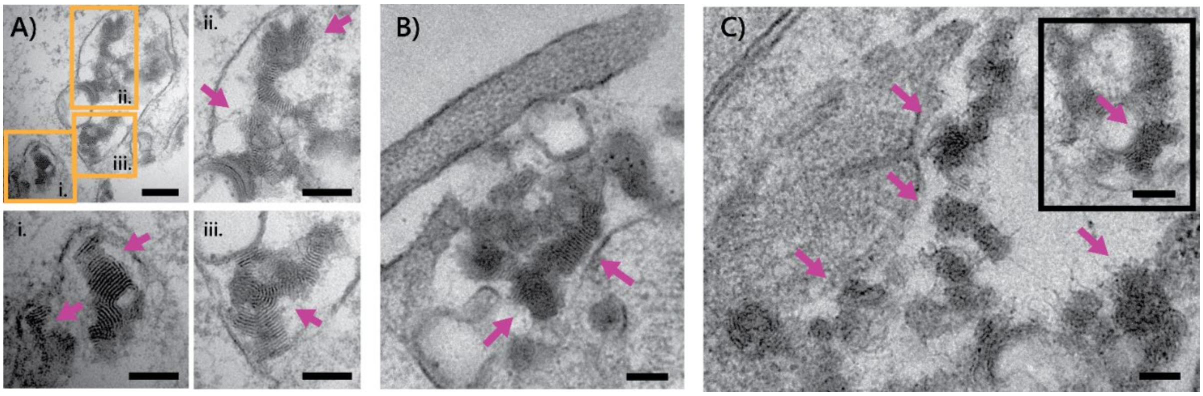

Figure S1. cmRNA and pDNA lipoplexes show ordered multilamellar structures. TEM photomicrographs of ultrathin sections of lipoplexes being internalized by cells show characteristic multilamellar structures of lipid complexes loaded with nucleic acids with periodic striations. (A,B) Metluc cmRNA + 3DFect. (C) Metluc pDNA + 3DFect.

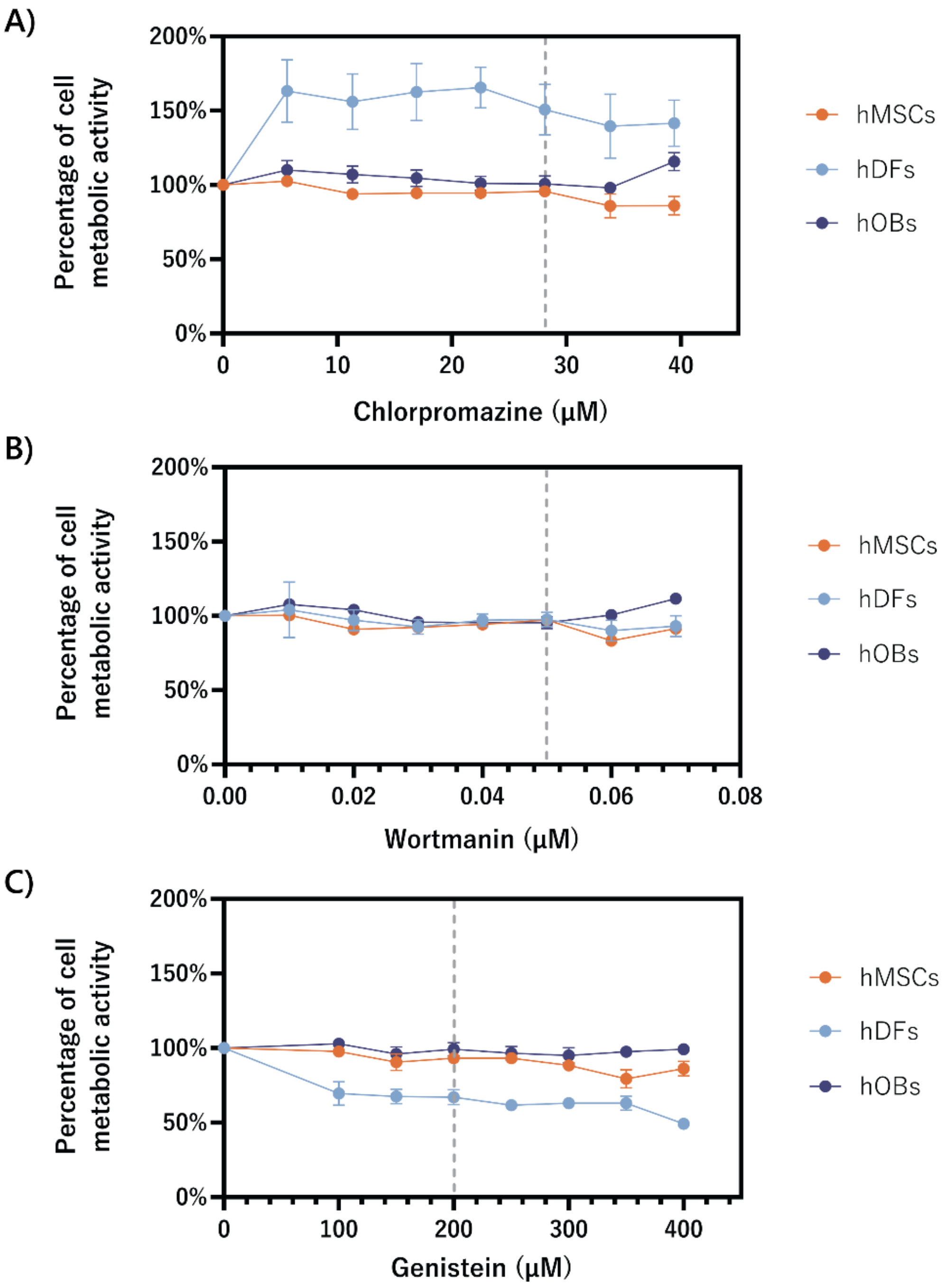

Figure S2. Percentage of cell metabolic activity as cytotoxic indicator of hMSCs, hDFs, and hOBs treated with endocytosis inhibitors. Metabolic activity was assessed using the commercially available reagent PrestoBlue, which is based on the reduction of resazurin to the fluorescent resorufin in viable cells. (A) Concentrations from 0-40 µM of chlorpromazine (an inhibitor of clathrin-mediated endocytosis) were tested. (B) Concentrations from 0-0.07 µM of wortmannin (an inhibitor of macropinocytosis) were tested. (C) Concentrations from 0-400 µM of genistein (an inhibitor of caveolae mediated endocytosis) were tested. Dotted lines indicate the chosen concentrations used on the next experiments (Fig. 2-4).

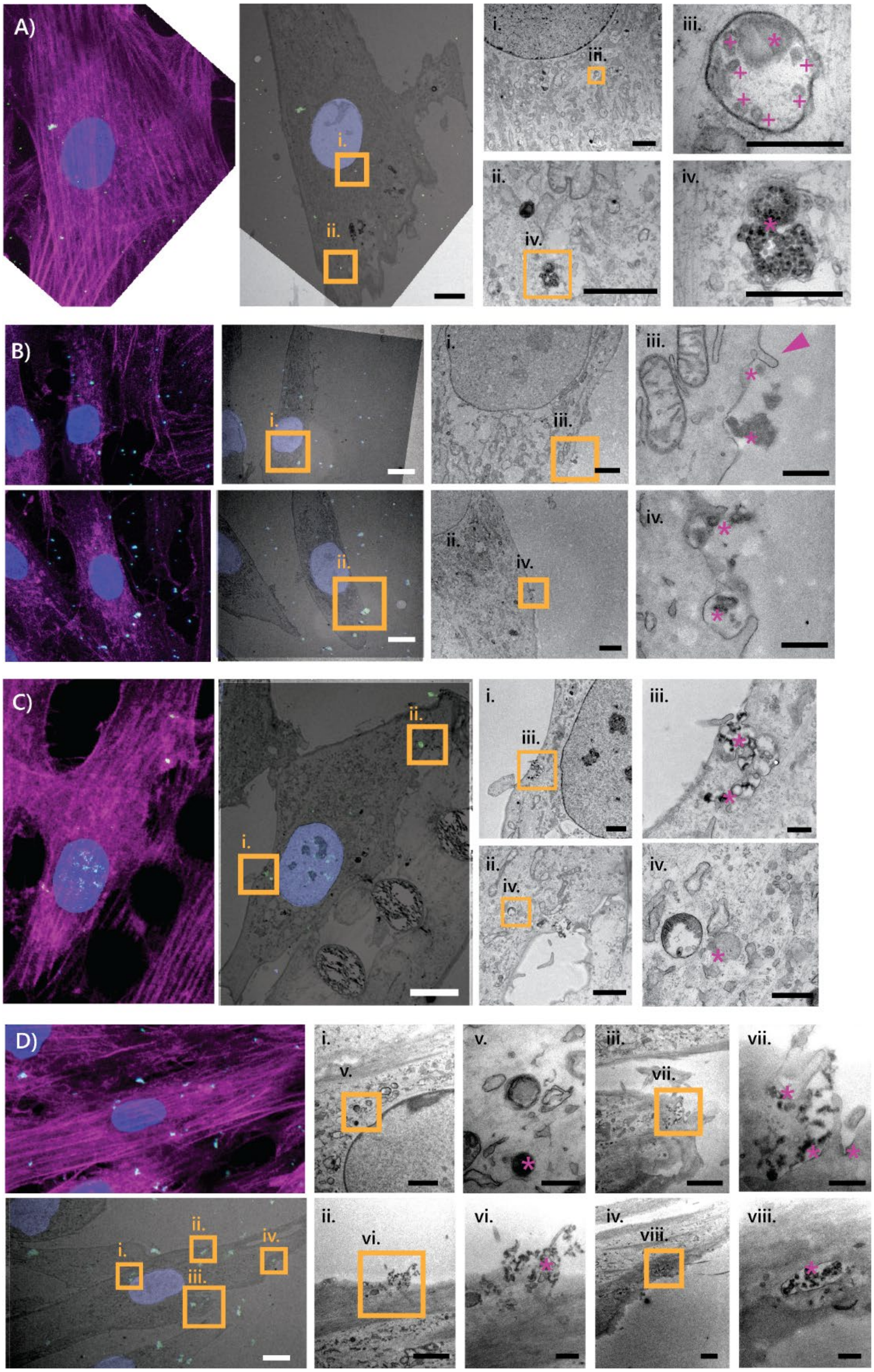

Figure S3. CLEM of hMSCs transfected with MFP-488 labelled MetLuc cmRNA or pDNA lipoplexes. Nuclear staining with Hoechst is shown in blue, actin filaments stained with phalloidin in magenta, and MFP-488 labelled lipoplexes are in green. Transfection of hMSCs was done with (A) cmRNA + Lipo complexes, (B) pDNA + Lipo complexes, (C) cmRNA + 3DFect complexes and (D) pDNA + Lipo complexes. (i-vi) are ultrathin sections representing high magnified regions of interest framed in yellow in the correlated image. (*) Depicts the specific localization of the internalized fluorescent complexes. Arrows point towards small flask-shaped invaginations of the plasma membrane. Scale bars in correlated images: 10 µm; in (i-ii): 2 µm and in magnified areas (iii-vi): 500 nm.

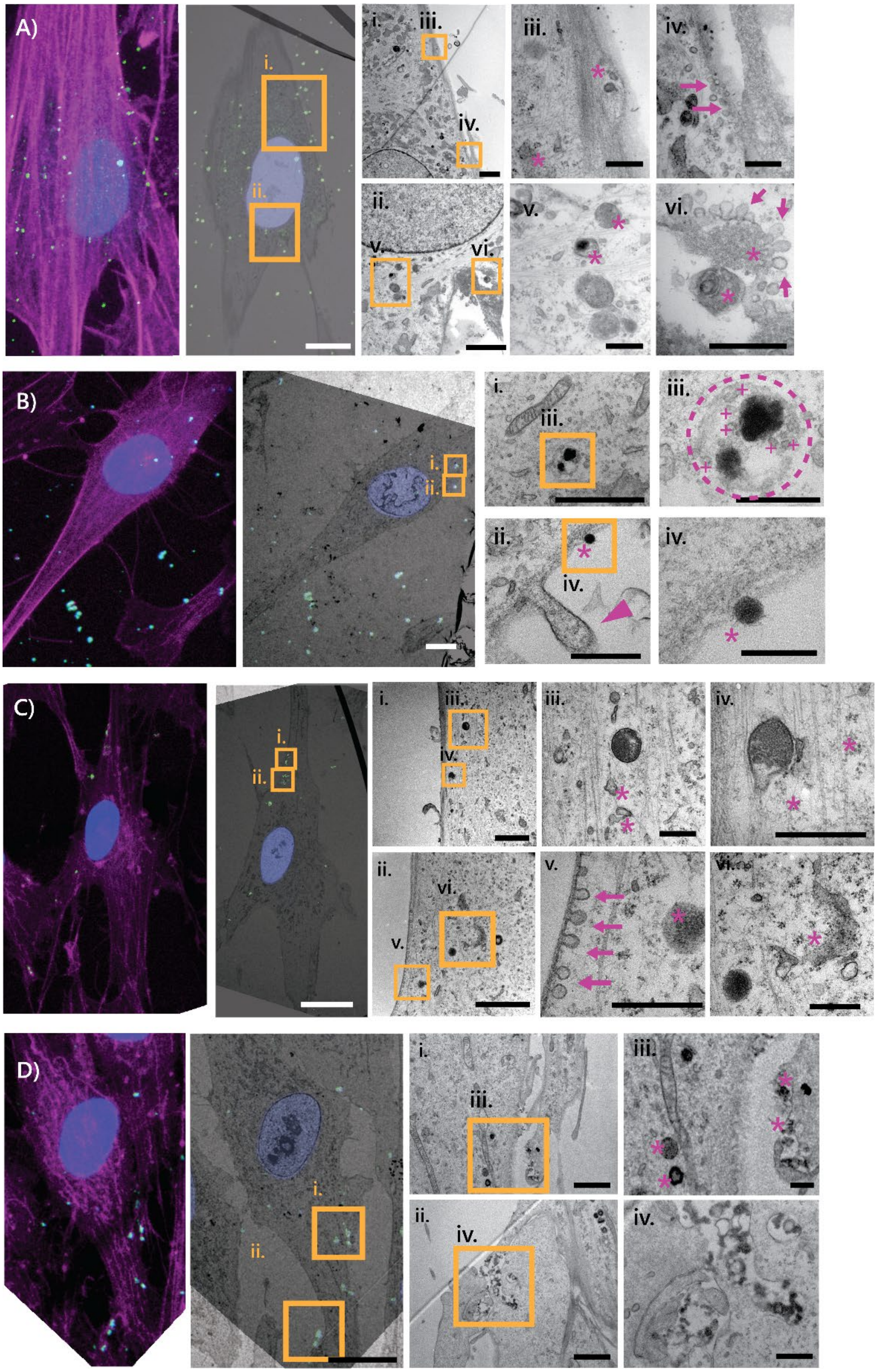

Figure S4. CLEM of hDFs transfected with MFP-488 labelled MetLuc cmRNA or pDNA lipoplexes. Nuclear staining with Hoechst is shown in blue, actin filaments stained with phalloidin in magenta, and MFP-488 labelled lipoplexes are in green. Transfection of hDFs was done with (A) cmRNA + Lipo complexes, (B) pDNA + Lipo complexes, (C) cmRNA + 3DFect complexes and (D) pDNA + 3DFect complexes. (i-vi) are ultrathin sections representing high magnified regions of interest framed in yellow in the correlated image. (*) Depicts the specific localization of the internalized fluorescent complexes. Arrows point towards small flask-shaped invaginations of the plasma membrane. Scale bars in correlated images: 10 µm; in (i-ii): 2 µm and in magnified areas (iii-vi): 500 nm.

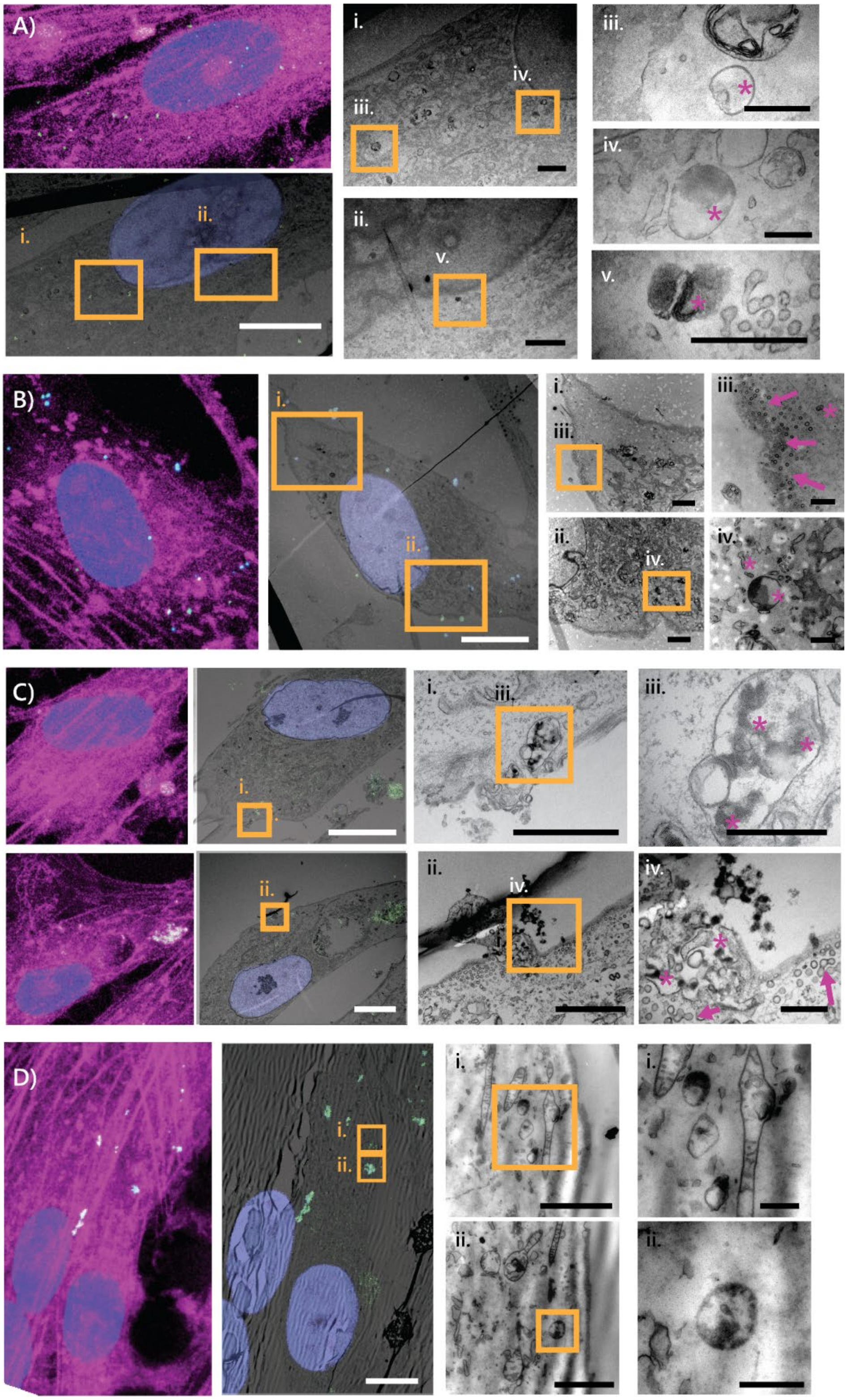

Figure S5. CLEM of hOBs transfected with MFP-488 labelled MetLuc cmRNA or pDNA lipoplexes. Nuclear staining with Hoechst is shown in blue, actin filaments stained with phalloidin in magenta, and MFP-488 labelled lipoplexes are in green. Transfection of hOBs was done with (A) cmRNA + Lipo complexes, (B) pDNA + Lipo complexes, (C) cmRNA + 3DFect complexes and (D) pDNA + Lipo complexes. (i-vi) are ultrathin sections representing high magnified regions of interest framed in yellow in the correlated image. (*) Depicts the specific localization of the internalized fluorescent complexes. Arrows point towards small flask-shaped invaginations of the plasma membrane. Scale bars in correlated images: 10 µm; in (i-ii): 2 10 µm and in magnified areas (iii-vi): 500 nm.

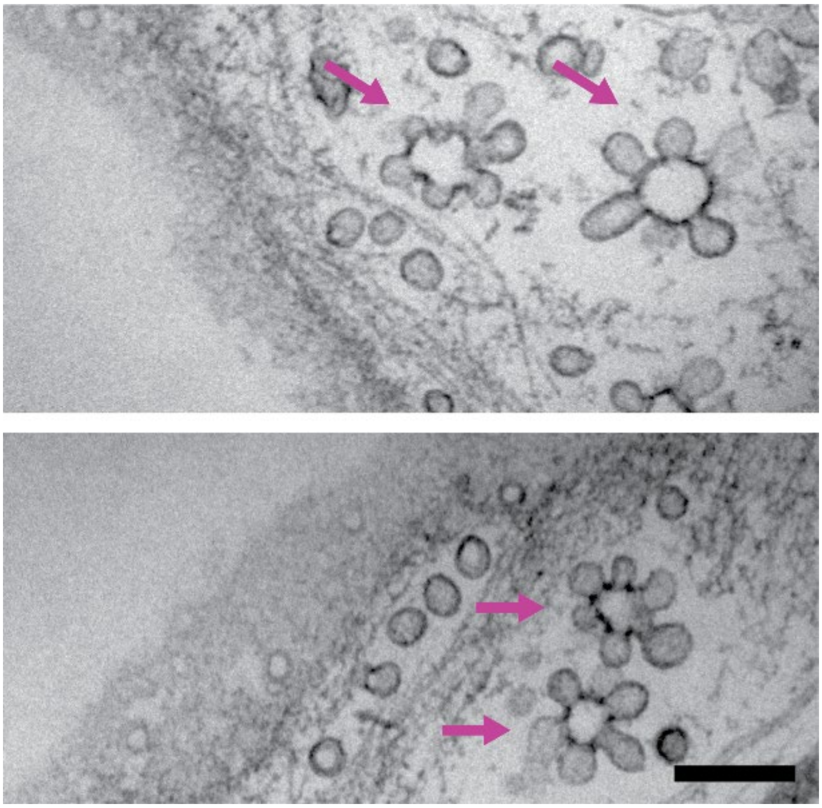

Figure S6. TEM photomicrographs of ultrathin sections reveals caveosomes (magenta arrows) as rosette-shaped structures in hOBs transfected with Metluc cmRNA + 3DFect complexes.

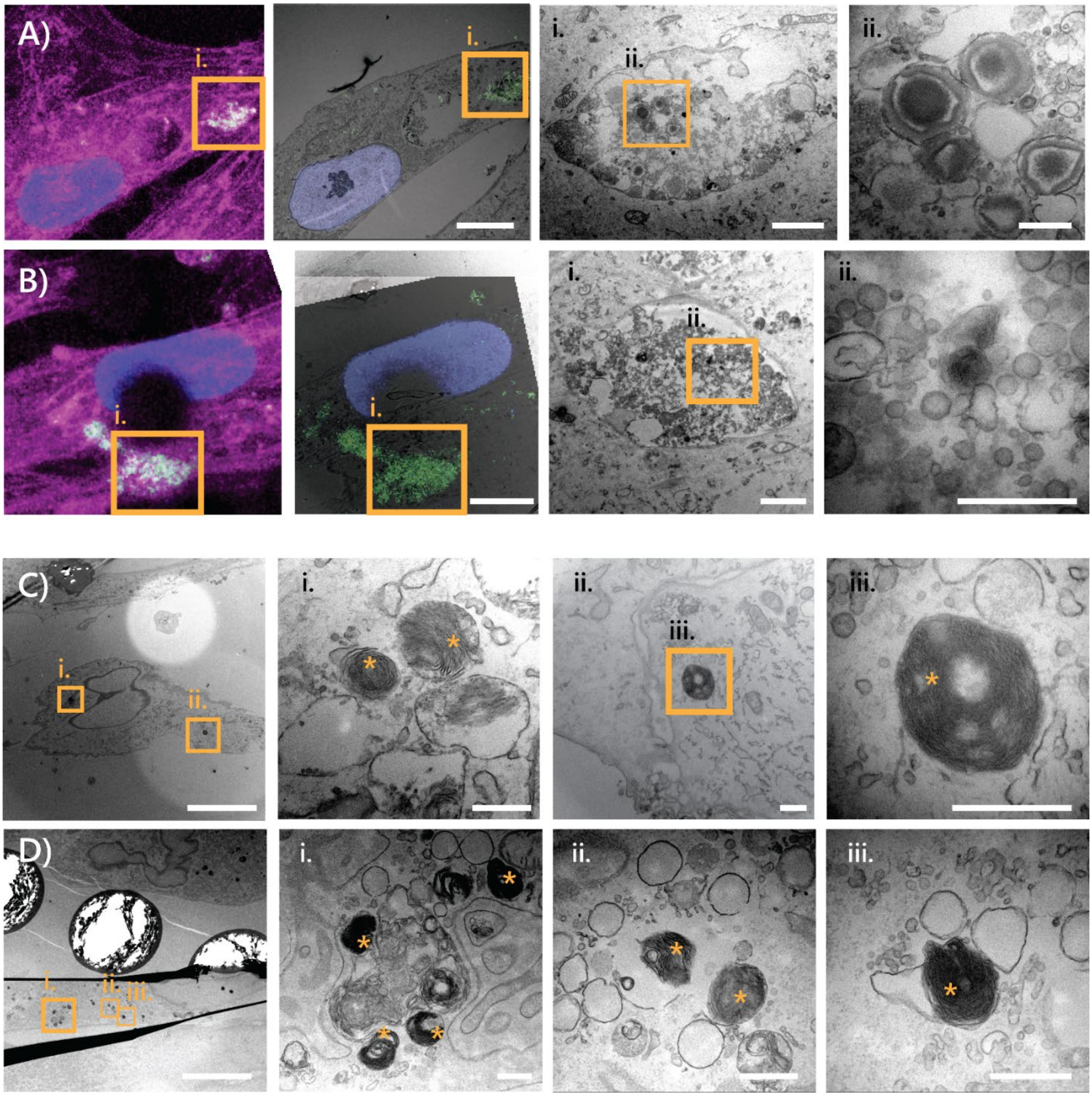

Figure S7. Phagosome-like structures colocalized with complexes in hOBs. (A and B) Fluorescence microscopy, CLEM and TEM images of hOBs transfected with labelled MetLuc lipoplexes. Nuclear staining with Hoechst is shown in blue, actin filaments stained with phalloidin in magenta, and MFP-488 labelled lipoplexes are in green. Yellow squares frame phagosome like-strucutres which can be better visualized in TEM high magnified regions (i-ii). (C and D) Lysosomal organelles containing multiple concentric membrane layers, characteristic of multilamellar bodies Scale bars in correlated images and in cell overviews in C and D: 10 µm; in (A-i and B-i): 2 µm; (A-ii and B-ii, C, i-iii and D, i-iii): 500 nm.

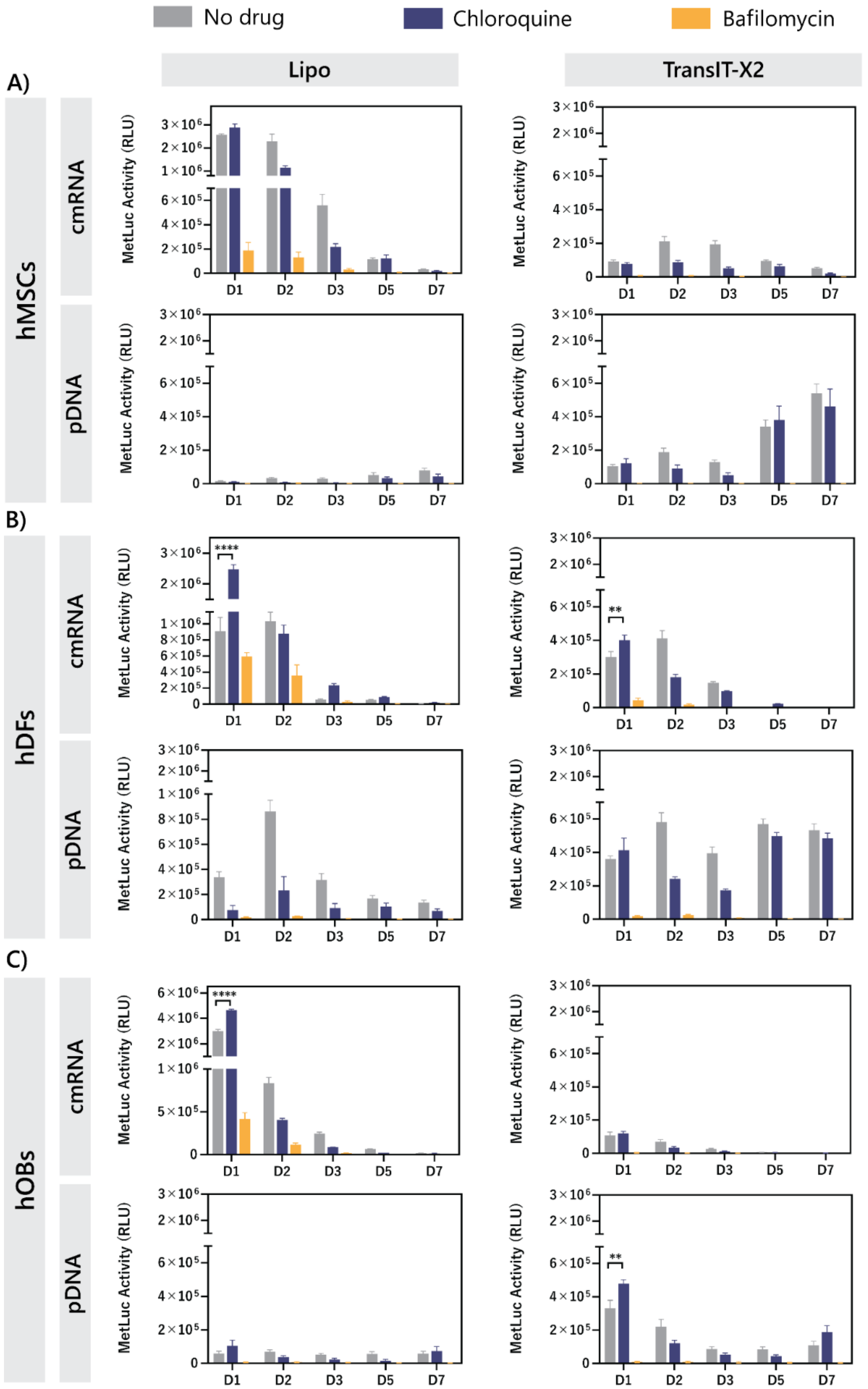

Figure S8. Effect on MetLuc activity after treatment with enhancers or inhibitors of endosomal escape. Transfection efficiencies over a time course of 7 days were determined by luminescent measurements after transfection in the presence or absence of the drugs chloroquine or bafilomycin in (**A**) hMSCs, (**B**) hDFs, and (**C**) hOBs, using MetLuc cmRNA or pDNA complexes with Lipo or TransIT-X2. Data are presented as mean ± SD (n ≥ 4). Abbreviations are explained as follows: MetLuc for Metridia luciferase, hMSCs for human mesenchymal stromal cells, hDFs for human dermal fibroblasts, hOBs for human osteoblasts, Lipo for Lipofectamine™3000, and RLU for Relative light units.

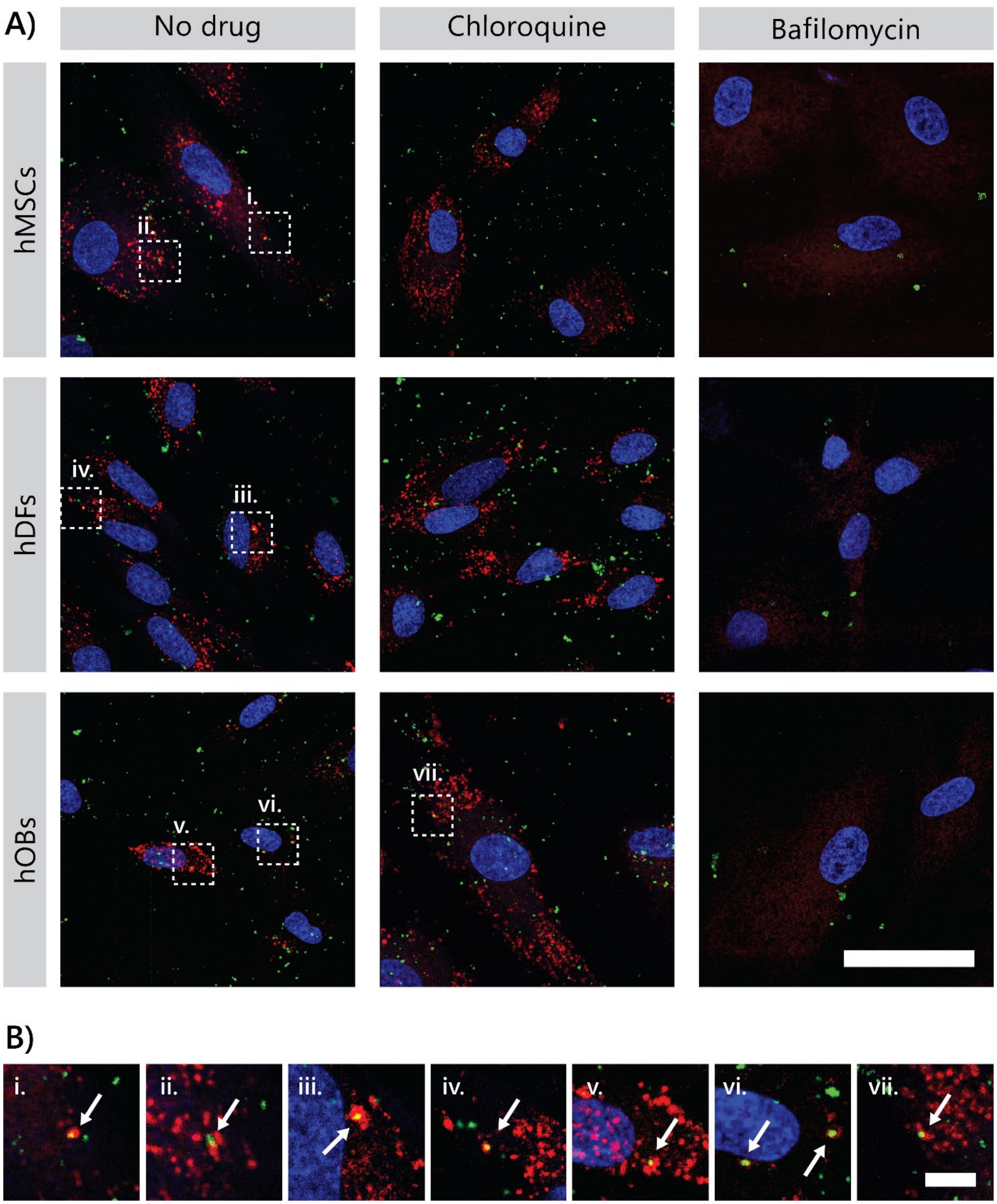

Figure S9. Lysosome trafficking of Lipofectamine™3000 complexes after treatment with enhancers or inhibitors of endosomal escape. (**A**) Fluorescence microscopy images of hMSCs, hDFs, and hOBs after 3 hours post-transfection with MFP-488 labelled complexes of Lipofectamine™3000 in the presence or absence of the drugs chloroquine or bafilomycin. LysoTracker™ Deep Red stained acidic compartments in red, nuclear staining with Hoechst is shown in blue and MFP-488 labelled complexes in green. Dashed squares (i-vii) indicate ROIs with colocalized complexes and lysosomes. (**B**) Zoomed-in images with arrows that point towards colocalized complexes. Abbreviations are explained as follows: hMSCs for human mesenchymal stromal cells, hDFs for human dermal fibroblasts, hOBs for human osteoblasts. Scale bars: (A) 50 µm; (B) 5 µm.

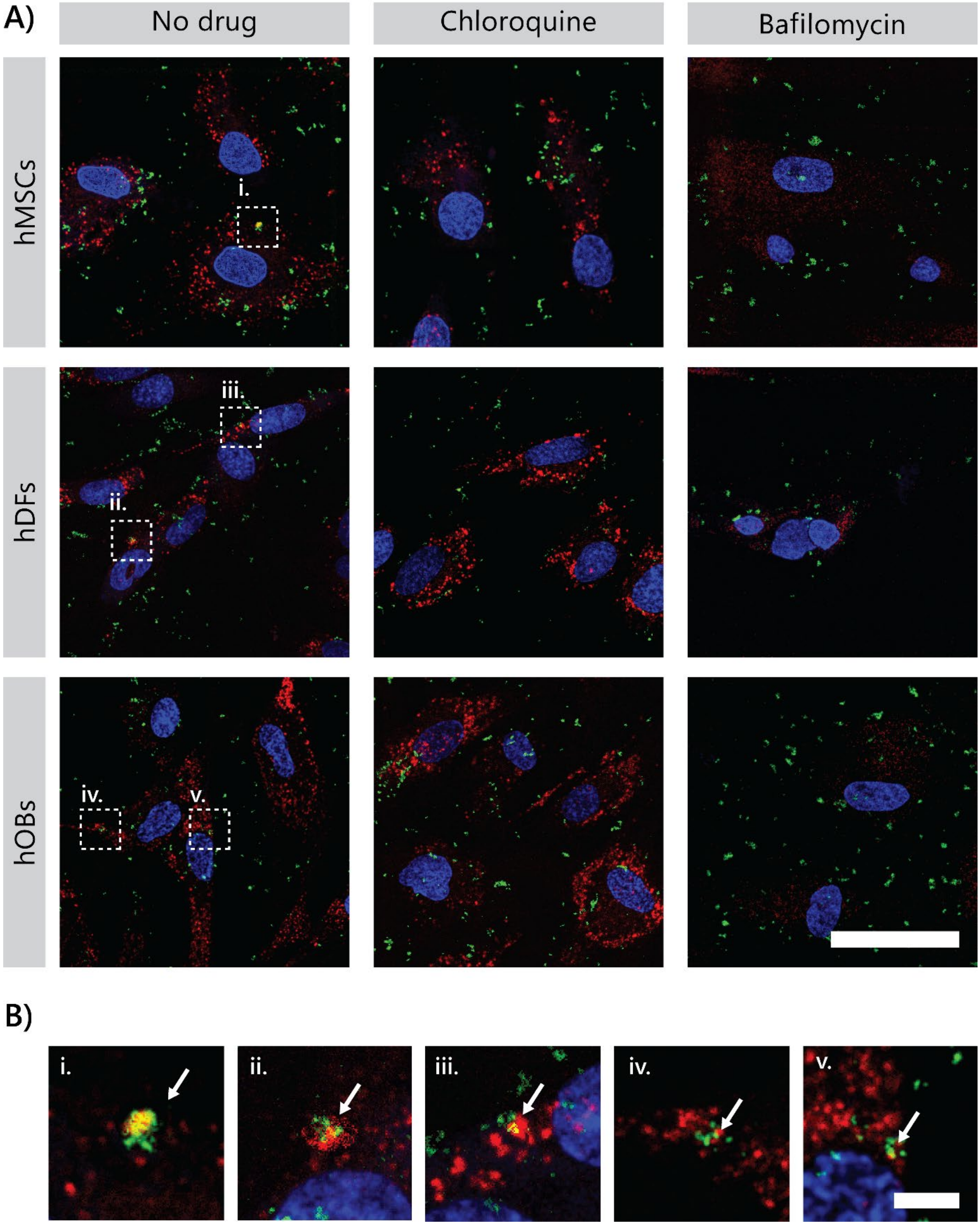

Figure S10. Lysosome trafficking of TransIT-X2 complexes after treatment with enhancers or inhibitors of endosomal escape. (**A**) Fluorescence microscopy images of hMSCs, hDFs, and hOBs after 3 hours post-transfection with MFP-488 labelled complexes of TransIT-X2 in the presence or absence of the drugs chloroquine or bafilomycin. LysoTracker™ Deep Red stained acidic compartments in red, nuclear staining with Hoechst is shown in blue and MFP-488 labelled complexes in green. Dashed squares (i-v) indicate ROIs with colocalized complexes and lysosomes. (**B)** Zoomed-in images with arrows that point towards colocalized complexes. Abbreviations are explained as follows: hMSCs for human mesenchymal stromal cells, hDFs for human dermal fibroblasts, hOBs for human osteoblasts. Scale bars: (A) 50 µm; (B) 5 µm.

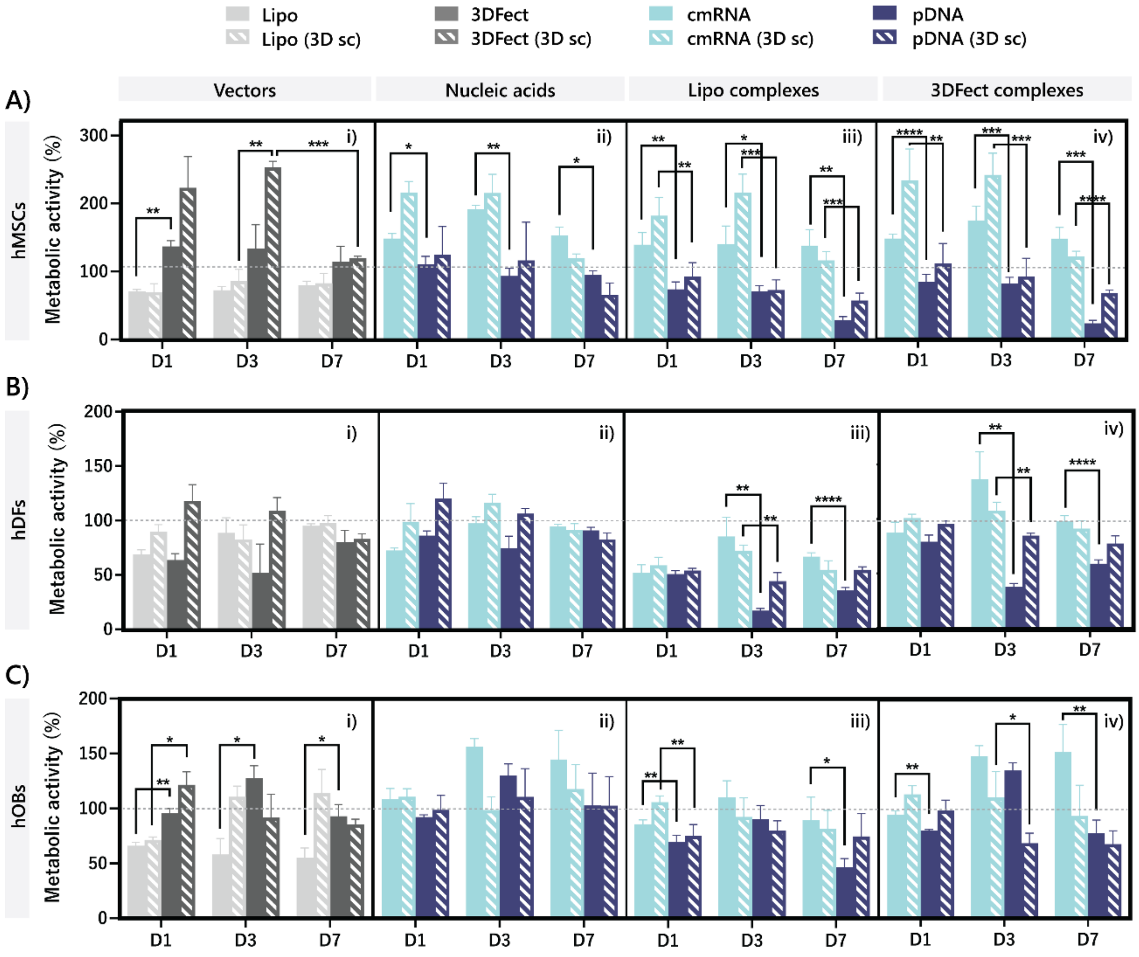

Figure S11. Percentage of cellular metabolic activity as an indicator of cytotoxicity after transfection. All complexes were formulated at a nucleic acid: vector ratio (w/w) of 1:2. Nucleic acids used code for the protein MetLuc. Percentages were calculated relative to untransfected cells (dotted grey lines). Complete bars represent cells seeded on standard treated well plates. Dashed lines represent cells seeded on ES scaffolds. Metabolic activity of bars in grey (i) show cells that received the delivery vectors alone, (ii) of cells treated with only the nucleic acids, (iii) of cells treated with Lipofectamine 3000 complexes, and (iv) of cells treated with 3DFect complexes. Bars in light blue and dark blue represent complexes of MetLuc cmRNA and MetLuc pDNA, respectively. (A) Human mesenchymal stromal cells. (B) Human dermal fibroblasts (C) Human osteoblasts. Each value represents the mean ± SD, (n=5).

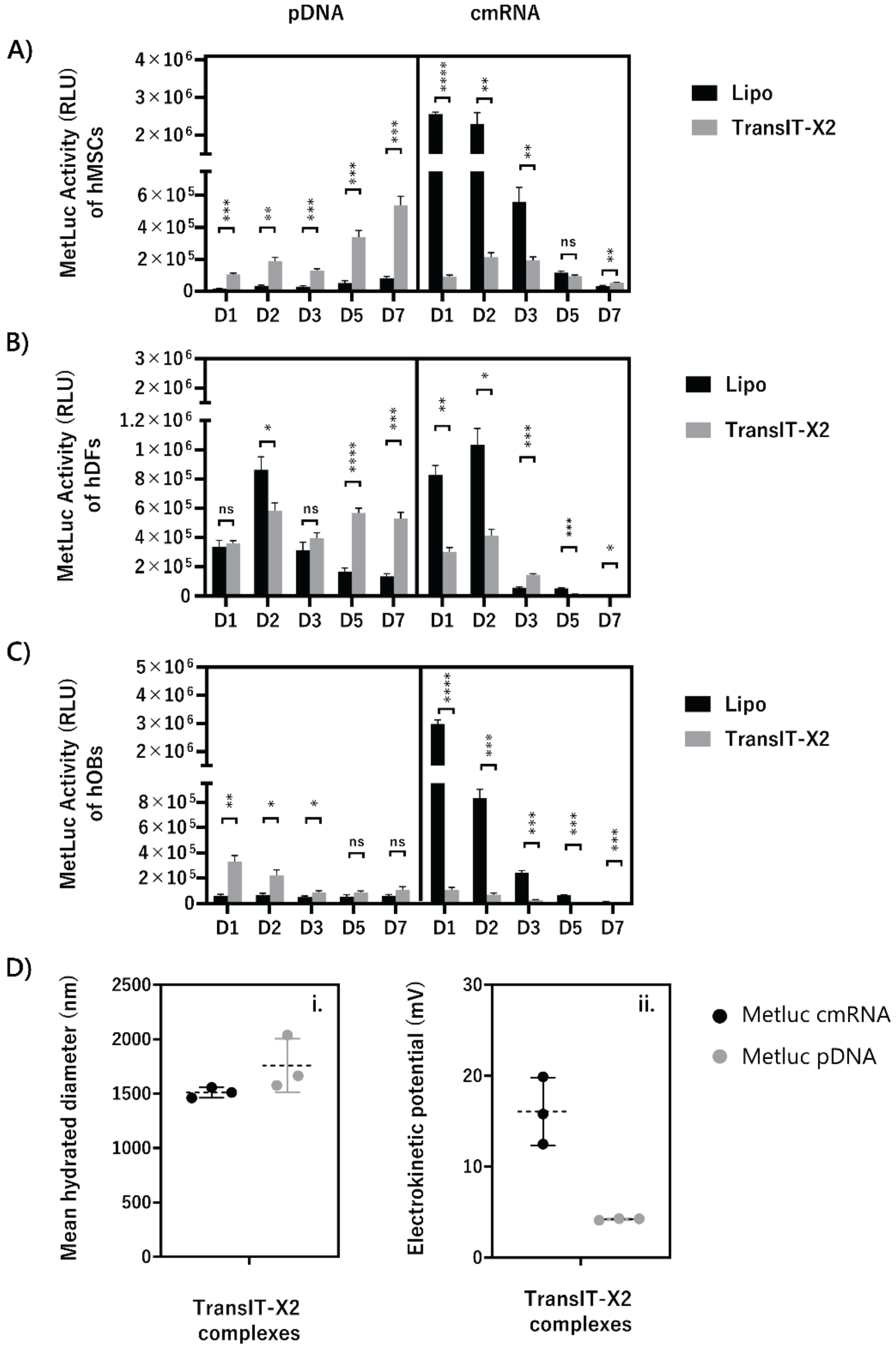

Figure S12. Comparison of MetLuc expression between lipid and polymeric vectors. Transfection efficiency is indicated as RLU over a time course of 7 days in (**A**) hMSCs, (**B**) hDFs, and (**C**) hOBs. Cells were transfected with either pDNA or cmRNA both encoding for MetLuc. The selected lipid vector was Lipofectamine™3000 (black bars) and the polymeric vector was TransIT-X2 (grey bars). Data are presented as mean ± SD (n = 4). Multiple comparisons were analyzed using two-way ANOVA with Sidak's correction. Of note, the y-axis in (A), (B), and (C) differ. (**D**) Characterization of the polymeric TransIT-X2 complexes with MetLuc cmRNA or pDNA. (i) Size displayed as mean hydrated diameter and (**B**) electrokinetic potential. Abbreviations are explained as follows: RLU for Relative light units, hMSCs for human mesenchymal stromal cells, hDFs for human dermal fibroblasts, hOBs for human osteoblasts, MetLuc for Metridia luciferase, and Lipo for Lipofectamine™3000.

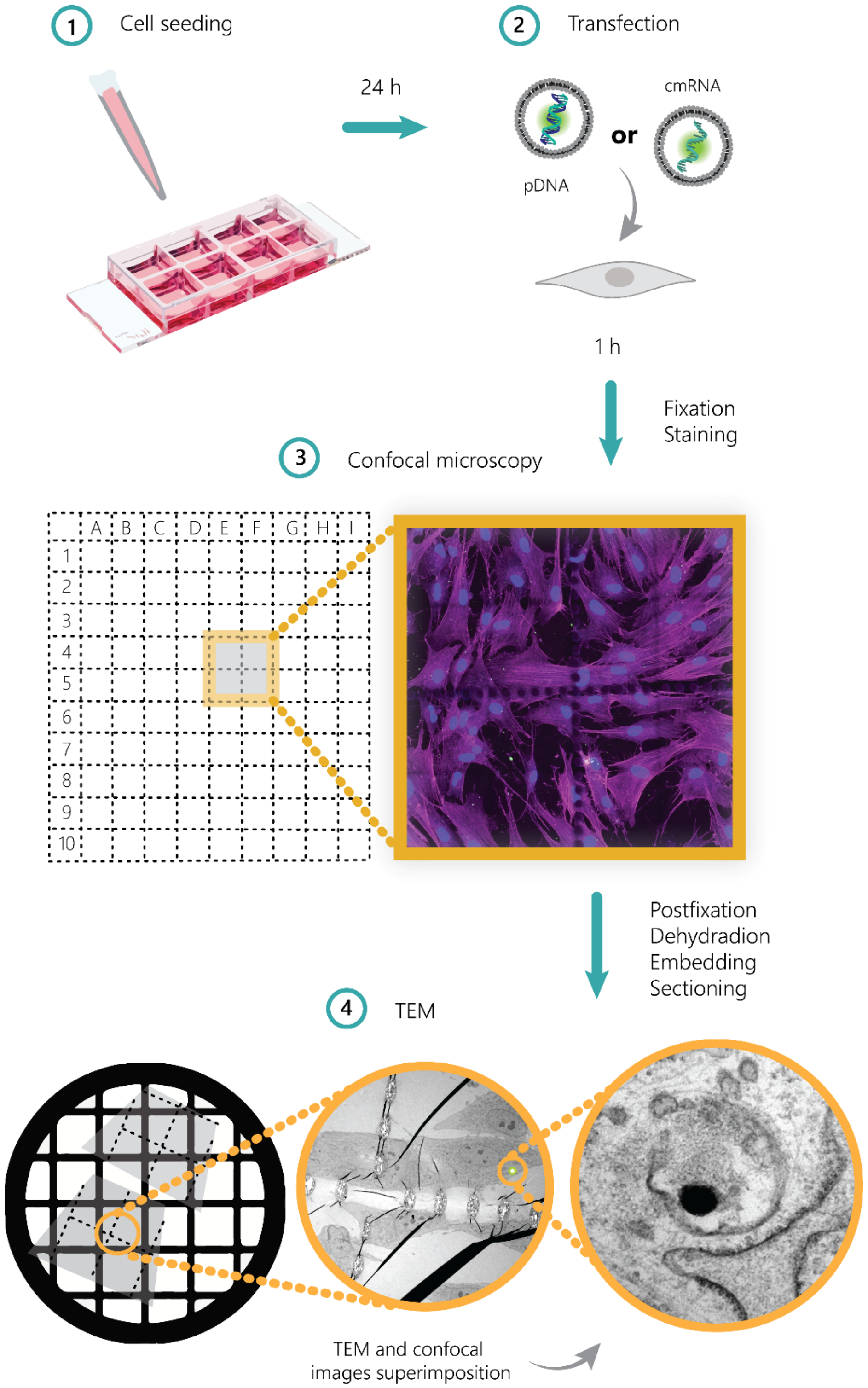

Figure S13. Procedure for CLEM imaging. hMSCs, hDFs, or hOBs were seeded on a µ-Slide 8 Well culture dishes. After 24 hours, cells were transfected with MFP488-Metluc pDNA or MFP488-MetLuc cmRNA (using either Lipofectamine™3000 or 3DFect). A region of interest was selected based on the imprinted coordinate system and imaged with confocal microscopy. Sample preparation for TEM included postfixation with buffer containing 1% osmium tetroxide and 1.5% potassium ferricyanide (K3[Fe(CN)6], dehydration in a graded series of ethanol, Epon resin infiltration and embedding, and sectioning.

Video S1. Trafficking of complexes to lysosomal compartments. 2 hour time-lapse video of internalized of 3DFect complexes colocalized with lysosomes in human osteoblasts. LysoTracker™ Deep Red stained acidic compartments in red, nuclear staining with Hoechst is shown in blue, and MFP-488 labelled MetLuc complexes in green.

REFERENCES

1. Wang, L.-H., Rothberg, K.G., and Anderson, R. (1993). Mis-assembly of clathrin lattices on endosomes reveals a regulatory switch for coated pit formation. Journal of Cell Biology 123, 1107-1117.

2. Thelen, M., Wymann, M.P., and Langen, H. (1994). Wortmannin binds specifically to 1-phosphatidylinositol 3-kinase while inhibiting guanine nucleotide-binding protein-coupled receptor signaling in neutrophil leukocytes. Proceedings of the National Academy of Sciences 91, 4960-4964.

3. Parton, R.G., Joggerst, B., and Simons, K. (1994). Regulated internalization of caveolae. Journal of Cell Biology 127, 1199-1215.
